# Dynamic changes in *cis*-regulatory occupancy by Six1 and its cooperative interactions with distinct cofactors drive lineage-specific gene expression programs during progressive differentiation of the auditory sensory epithelium

**DOI:** 10.1093/nar/gkaa012

**Published:** 2020-01-20

**Authors:** Jun Li, Ting Zhang, Aarthi Ramakrishnan, Bernd Fritzsch, Jinshu Xu, Elaine Y M Wong, Yong-Hwee Eddie Loh, Jianqiang Ding, Li Shen, Pin-Xian Xu

**Affiliations:** 1 Department of Genetics and Genomic Sciences, Icahn School of Medicine at Mount Sinai, New York, NY 10029, USA; 2 Department of Neurosciences, Icahn School of Medicine at Mount Sinai, New York, NY 10029, USA; 3 Department of Biology, University of Iowa, Iowa, IA 52242-1324; 4 Department of Infectious Diseases, Shunde Hospital, Southern Medical University, Shunde 528308, Guangdong, China; 5 Department of Cell, Developmental and Regenerative Biology, Icahn School of Medicine at Mount Sinai, New York, NY 10029, USA

## Abstract

The transcription factor Six1 is essential for induction of sensory cell fate and formation of auditory sensory epithelium, but how it activates gene expression programs to generate distinct cell-types remains unknown. Here, we perform genome-wide characterization of Six1 binding at different stages of auditory sensory epithelium development and find that Six1-binding to *cis*-regulatory elements changes dramatically at cell-state transitions. Intriguingly, Six1 pre-occupies enhancers of cell-type-specific regulators and effectors before their expression. We demonstrate in-vivo cell-type-specific activity of Six1-bound novel enhancers of *Pbx1*, *Fgf8*, *Dusp6*, *Vangl2*, the hair-cell master regulator *Atoh1* and a cascade of *Atoh1*’s downstream factors, including *Pou4f3* and *Gfi1*. A subset of Six1-bound sites carry consensus-sequences for its downstream factors, including Atoh1, Gfi1, Pou4f3, Gata3 and Pbx1, all of which physically interact with Six1. Motif analysis identifies RFX/X-box as one of the most significantly enriched motifs in Six1-bound sites, and we demonstrate that Six1-RFX proteins cooperatively regulate gene expression through binding to SIX:RFX-motifs. Six1 targets a wide range of hair-bundle regulators and late *Six1* deletion disrupts hair-bundle polarity. This study provides a mechanistic understanding of how Six1 cooperates with distinct cofactors in feedforward loops to control lineage-specific gene expression programs during progressive differentiation of the auditory sensory epithelium.

## INTRODUCTION

The transcription factor (TF) Six1 belongs to the sine oculis (So/Six) homeobox protein family that plays important roles in the development of multiple organs, including ear, urinary system and kidney (1–4). Overexpression of SIX1 is associated with many human cancers (5), while mutations in the human *SIX1* cause Branchio-Oto-Renal (BOR) or Branchio-Oto (BO) syndrome (6). Approximately 93% of BOR/BO patients exhibit hearing loss, which can be conductive, sensorineural or a combination of both due to malformations of outer, middle and/or inner ear (7,8). The mammalian inner ear sensory organ for hearing—the organ of Corti—in the cochlea houses two types of hair cells: one row of inner and three rows of outer hair cells interdigitated with several subtypes of supporting cells—one inner border, one inner phalangeal, inner and outer pillar, and three rows of Deiters' cells aligned in a medial-to-lateral direction, all of which differentiate from common precursors (9–11). Failure to generate or maintain these epithelial cells in the organ of Corti causes irreversible deafness due to lack of regenerative capacity of the cochlea. However, developmental programs that generate these distinct subtypes are not understood, thus presenting a major challenge for clinical applications of guided cell differentiation strategies to replace lost hair cells.

During differentiation, the precursors acquire distinct molecular, anatomical, and functional properties, a process dictated by combinations of lineage- and subtype-specific genes. TFs are crucial to this cellular complexity and act in a combinatorial fashion to control the network of lineage-specific gene expression programs by binding to their DNA-binding motifs present in the *cis*-regulatory elements (CREs) of genes. In order to regenerate hair cells after damage by altering differentiation programs that control cellular states in the sensory epithelium, we must understand the causal relationship between TF activities and cellular phenotypes. The TF Six1 is critical for neurosensory cell development and *Six1^−^^/^^−^* mice lack neurosensory structures of the inner ear (12,13). Conversely, forced expression of Six1 with the phosphatase-transcriptional coactivator Eya1 in cochlear explants converts nonsensory cochlear cells to either hair cells (14) or spiral ganglion neurons in combination with the chromatin-remodeling complex Brg1-BAFs (15). Recent analyses of *Six1* conditional deletion in undifferentiated progenitors revealed that Six1 regulates hair cell fate induction and auditory sensory epithelium formation (16). However, it remains unclear whether Six1 also plays a role in mediating hair cell differentiation after fate induction. Furthermore, Six1-bound CREs and its genome-wide gene targets or cell- or stage-specific cofactors necessary for Six1’s activity in controlling lineage-specific expression programs in the inner ear are unknown.

Here, we characterized Six1-binding properties over a period from cell-cycle exit of prosensory progenitors to hair cell stereociliary bundle development during differentiation. Six1 reveals dynamic changes in its binding pattern during cell-state transition and pre-occupies CREs of a wide range of regulators necessary for both hair and supporting cell differentiation before their expression, many of which form protein complexes with Six1. Motif analysis revealed a novel combinatorial interaction of Six1 with RFX cofactors, as consensus-sequences for RFX/X-box was identified as one of the most significantly enriched motifs in a subset of Six1 CREs. We demonstrate that Six1 and Rfx1/3 cooperatively regulate gene expression through binding to SIX:RFX-motifs and that cell-type-specific activity of multiple CREs/enhancers at key loci and their Six1-dependent expression in vivo. Late deletion of *Six1* disrupts both hair-bundle structure and orientation. We also identify a broad set of CREs/enhancers of a wide range of planar-cell-polarity and hair-bundle regulators, of which 83 contain mutations known to cause human deafness syndromes. Intriguingly, Six1 pre-occupies CREs of hair or supporting cell subtype-specific effectors in undifferentiated precursors. Our findings provide a mechanistic understanding of how Six1 changes occupancy during auditory sensory epithelium development and interacts with differentially expressed downstream TFs and signaling pathways to not only initiate cell fate induction but also mediate sequential differentiation to progressively restrict the identity of distinct cell-types. This study represents the first systematic characterization of Six1-controlled transcriptional networks in inducing cell diversification, differentiation and hair-bundle formation in the auditory sensory epithelium.

## MATERIALS AND METHODS

### Mice and tamoxifen treatment

Wild-type, *Eya1^CreERT2^* (17) and *Six1^fl^* (16) mice were used following the animal protocols (06-0807), which was approved by the Animal Care and Use Committee (ACUC) at the Icahn School of Medicine at Mount Sinai.

For induction of the CreER protein, tamoxifen (T5648, Sigma) was dissolved in corn oil (C8267, Sigma) and administrated (1.5 mg/10 g body weight) by oral gavage.

### ChIP-seq and quantitative real-time ChIP-PCR

For Six1 ChIP, 50 (E13.5) to 40 (E16.5) cochleae were used, while 10 cochleae were sued for H3K29ac or H3K27me3 ChIP. Cochleae were dissected from E13.5, E15.5 or E16.5 wild-type embryos and cochlear epithelia were dissected from cochleae, which also contained surrounding mesenchyme tissues, like we did previously for cochlear explant culture (14). The dissected epithelia were cross-linked with 1% formaldehyde at room temperature for 30 mins, and then homogenized and lysed in cold lysis buffer (50 mM HEPES–KOH, pH 7.5, 140 mM NaCl, 1 mM EDTA, 10% glycerol, 0.5% NP-40, 0.25% Triton X-100, 1× protease inhibitors). Samples were pelleted at 2000 g at 4°C and resuspended in cold wash buffer (10 mM Tris–HCl, pH 8.0, 200 mM NaCl, 1 mM EDTA, 0.5 mM EGTA, 1× protease inhibitors) for 10 min in 15 ml conical tubes, followed by spinning at 2000 g at 4°C in a benchtop centrifuge. Samples were resuspended in 1 ml cold sonication buffer (10 mM Tris–Cl, pH 8.0, 2 mM EDTA, 0.1% SDS) and sonicated to 200–500 bp fragments using a Covaris S220 Focused-ultrasonicator. Sonicated chromatin was cleared by pelleting insoluble material at 13 000 RPM at 4°C, followed by preclear with protein A/G beads and incubation with 1–2 μg antibody overnight (anti-Six1, HPA001893, Sigma; anti-H3K27ac, ab4792, Abcam; anti H3K27me3, ab6002, Abcam) or 1–2 μg rabbit IgG as a negative control. Chromatin–antibody complexes were precipitated with protein A/G beads at 4°C for another 5 h. Immunoprecipitated complexes were subjected to series of wash steps with low salt buffer (20 mM Tris–Cl 8.0, 150 mM NaCl, 2 mM EDTA, 1% Triton X-100, 0.1% SDS), high salt buffer (20 mM Tris–Cl pH 8.0, 500 mM NaCl, 2 mM EDTA, 1% Triton X-100, 0.1% SDS), LiCl wash buffer (10 mM Tris–HCl pH 8.0, 250 mM LiCl, 1 mM EDTA, 1% NP-40, 1% sodium deoxycholate) and TE plus NaCl, followed by elution and reverse crosslinking overnight at 65°C. The quality controls of ChIPed DNA was performed with Qubit 2.0 Fluoremeter using dsDNA HS assay Kit (Q32854, ThermoFisher Scientific) and Agilent 2200 TapeStation System using High Sensitivity D1000 Reagents (5067–5585, Agilent). The libraries for sequencing were prepared with the ThruPLEX DNA-seq Kit (R400429, Rubicon Genomics) and sequenced on Illumina HiSeq 2500 system. The genomic input DNA was also used to prepare libraries and sequencing as controls for peaking calling.

For quantitative real-time ChIP-qPCR, chromatin derived from cochleae was precipitated with IgG, anti-Six1 (HPA001893, Sigma), -Rfx1 (sc-374270, Santa Cruz) or -Rfx3(HPA035689, Sigma) respectively. The ChIPed DNAs were subjected to quantitative real-time PCR (qPCR) amplification with StepOnePlus PCR system and SYBR green PCR Master Mix kit (4309155, Applied Biosystems). This experiment was repeated three times and each qPCR was performed in triplicate. The enrichment fold of IP over mock IP (IgG) was calculated using the comparative Ct (threshold cycle) method. IPs and mock IPs were normalized to inputs and the enrichment of mock IP was considered 1-fold. The Student's *t*-test was used to determine the significance of enrichment changes for the ChIP-qPCR experiments. Error bars indicates SEM. *n* = 3 independent experiments. *P < 0.05, **P < 0.01, ***P < 0.001 by two-tailed Student's *t*-test. The primers used for ChIP-qPCR are listed in Supplementary Table S1. The DNA positions are denoted relative to the transcriptional start site (+1).

For ChIP-qPCR using chromatin prepared from 293 cells, 293 cells were cotransfected with each reporter transgene driven by CRE or CRE carrying mutated SIX-motifs or SIX:RFX motifs in combination with empty pcDNA3.0 vector, His-Six1/pcDNA3.0 or Flag-Rfx3 expression plasmid alone or in combination. Cell fixation, chromatin preparation and ChIP assay were performed as described in the ChIP protocol above. Transfection was repeated three times and each qPCR was performed in triplicate.

### Peak calling, gene otology and motif analysis

The ChIP-seq data were first checked for quality using the various metrics generated by FastQC (v0.11.2) (http://www.bioinformatics.babraham.ac.uk/projects/fastqc). Raw sequencing reads were then aligned to the mouse mm10 genome using default settings of Bowtie (v2.2.0) (18). Only uniquely-mapped reads were retained and duplicates were removed. Peak-calling was performed using MACS (v2.1.1) (19) with various *P*-value cutoffs as reported in the main text. Both genomic input and IgG ChIP-seq controls were used for peak calling. The peak bed files were generated from peak calling against genomic input control or IgG control with the default setting. The common peaks from these two bed files were used for subsequent analyses. Motif enrichment analysis was performed using the Homer package (v4.8.3) (20). The peak annotation and gene ontology analysis was performed using GREAT program (21) and Panther classification system (22).

### Transgenic analysis of enhancer activity and site mutagenesis of Six1/2- and RFX-binding motifs in the enhancer reporters

The Hsp68 minimal promoter was inserted into pWhere vector (Invivogen) to drive LacZ or eGFP expression flanked by the H19 insulators and individual enhancer element was inserted upstream of the Hsp68 minimal promoter. Pronuclear injection was performed at our Mouse Genetics and Gene Targeting facility. Transgene expression was analyzed in G0 embryos at different stages.

Site-directed mutagenesis of Six1/2-binding sites or Six1/2:RFX motifs in combination in the enhancer sequences was performed to generate mutant reporters. The primers for site mutagenesis were listed in supplementary materials (Supplementary Table S1).

### Transfection and expression plasmids

Two hundred ninety-three cells were cultured and used for transfection as described previously (23). Reporter transgene plasmids used for transfection were constructed as described above.

Expression plasmid: His-Six1 pcDNA3.0(20) and pRRLHA-Atoh1 was constructed in our lab. Flag-Rfx3 (OMu17515D, GenScript), Flag-Gfi1 (MR227196, Origene), Flag-Pou4f3 (MR223064, Origene), Flag-Gata3 (MR227460, Origene) or Flag-Pbx1 (MR206861, Origene).

### Histology, immunohistochemistry, *in situ* hybridization and X-gal staining

Histology, Immunohistochemistry and *in situ* hybridization were performed as described previously (23). Average 5–6 embryos of each genotype were used for each experiment.

### Co-immunoprecipitation and western blot

Cochleae of E14.5, E15.5 or E17.5 or 293 cells transfected with His-Six1 and HA-Atoh1, Flag-Pou4f3, -Gfi1, -Rfx3 or -Pbx1 expression plasmids were lysed in homogenized and lysed in 10 mM HEPES, pH 7.9, 1.5 mM MgCl_2_, 10 mM KCl, 1 mM dithiothreitol and protease and phosphatase inhibitors cocktail. After removal of cytoplasmic fraction, the crude nuclei pellet was lysed in 20 mM HEPES, pH 7.9, 1.5 mM MgCl_2_, 420 mM NaCl, 0.2 mM EDTA, 25% glycerol, 1 mM DTT and protease and phosphatase inhibitors cocktail. The extracted nuclear proteins were diluted with IP buffer (20 mM Tris–HCl, pH 8.0, 150 mM NaCl, 0.1% NP-40, 10% glycerol), pre-cleared with protein A/G beads (sc-2003, Santa Cruz). After removal of the beads, the lysates were incubated with ∼1 μg primary antibodies overnight at 4°C and the protein–antibody complex were pulled down by adding 20 μl beads pre-blocked with BSA. The IPed protein complex was washed by IP buffer plus 0.2% NP40 for four times and analyzed, separated in SDS–PAGE and detected with differentiation primary antibodies and HRP-conjugated secondary using the enhanced chemiluminescence (ECL) method (WBKLS0500, Millipore).

Primary antibodies: anti-Six1 (HPA001893; Sigma), -Rfx1 (sc-374270, Santa Cruz), -Rfx3 (HPA035689, Sigma), -Flag (F7425, Sigma), -CTCF (ab70303, Abcam), -Gfi1 (sc-373960, Santa Cruz), -Pou4f3 (sc-81980, Santa Cruz), -Atoh1 (sc-136173, Santa Cruz), -Pbx1(ab97994, Abcam). The secondary antibody: anti-Rabbit IgG light chain (HRP) (ab99697, Abcam) and mouse IgG light chain binding protein m-IgGκ BP-HRP (sc-516102, Santa Cruz).

### Reverse transcription and real-time PCR

Cochlear epithelia were dissected from P0 inner ears and used for total RNA extraction using Trizol Reagents (Invitrogen). Total RNAs were treated with RNase-free DNase Set (QIAGEN) and then used for reverse transcription and PCR were performed as described previously using the Applied Biosystems StepOnePlus Real-Time PCR Systems (16). Expression levels of each transcript were normalized using β-actin as an internal control. Each set of experiments was repeated three times, and the ddCt relative quantification method (24,25) was used to evaluate quantitative variation. Two-tailed Student's *t* test was used for statistical analysis. Primers used are listed in Supplementary Table S1.

## RESULTS

### Dynamic changes in genomic occupancy by Six1 during auditory sensory cell fate commitment

**Figure 1.**
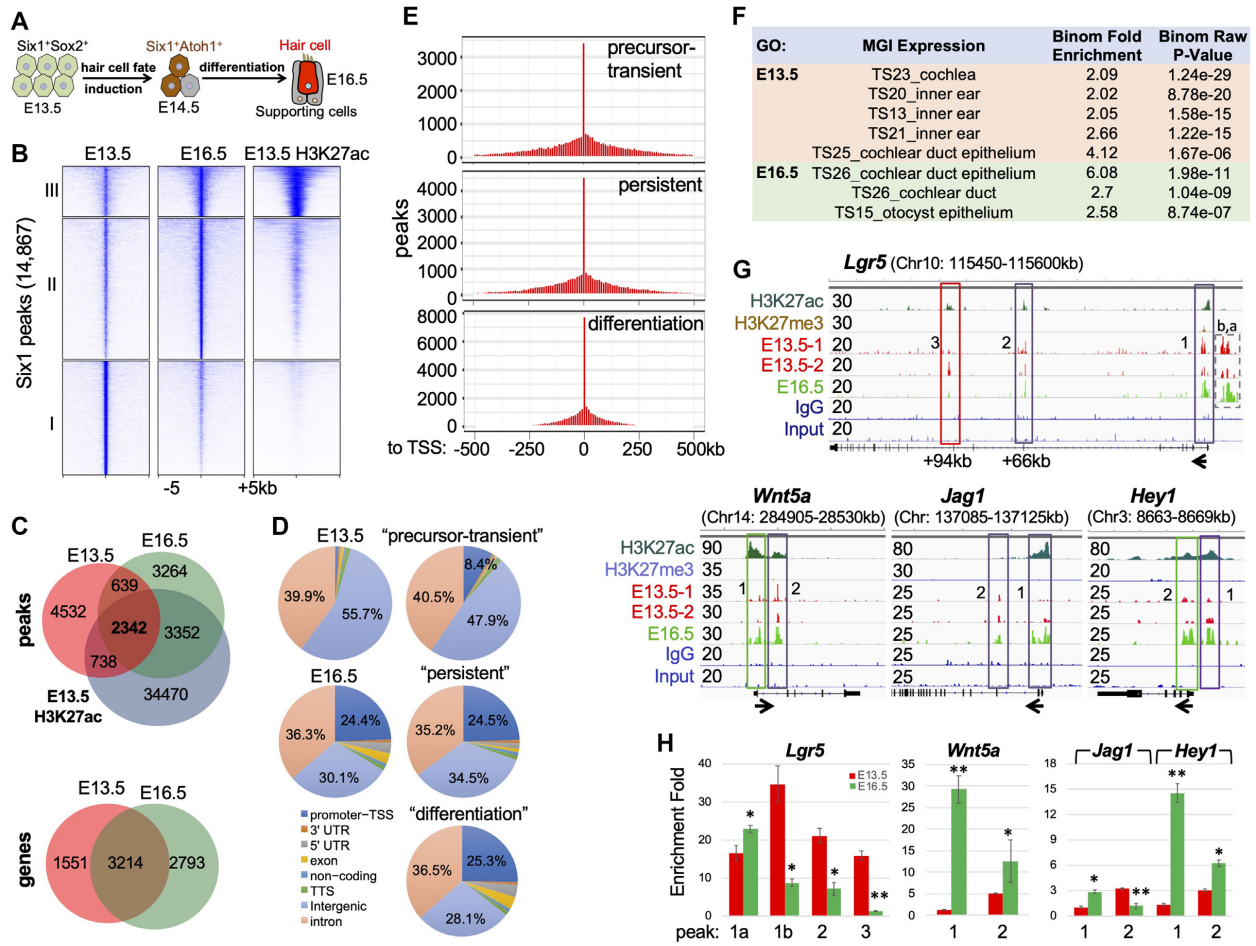
Six1 binding is dynamic across the transition of prosensory precursors to hair-bundle development. (**A**) Schematic drawing of time course of cochleae for ChIP-seq analysis. (**B**) Clustered heatmaps of Six1 and H3K27ac within a −5 kb/+ 5 kb window centered on all 14 867 Six1 peaks in E13.5 and E16.5 cochlea and overlapping with the deposition of H3K27ac in E13.5 cochlear epithelium. Peaks were called with the MACS program with a *P* value cut-off of 1e–5. (**C**) A Venn diagram indicating overlap of Six1-binding sites of E13.5 and E16.5 and of H3K27ac-deposition at E13.5. Lower panel indicating overlap of Six1-associated genes between E13.5 and E16.5. (**D**) Genomic distribution of Six1-enriched regions. (**E**) Distribution of Six1 peaks relative to TSSs. (**F**) GREAT analysis showing association of Six1-enriched regions with terms in the mouse gene expression information (MGI) database. (**G**) Genome browser visualization of Six1 peaks at *Lgr5*, *Wnt5a*, *Jag1* and *Hey1*. y-Axis numerical values in each track indicate track height scaling in read depth. The direction of transcription is shown by the arrow beginning at the TSS. (**H**) ChIP-qPCR analysis of the boxed peaks in (G) confirming stage-related changes in Six1-binding. IPs and mock IPs (IgGs) were normalized to inputs and the enrichment of mock IP was considered 1-fold (not shown). *P < 0.05, **P < 0.01.

We performed ChIP-seq to investigate the global occupancy of Six1-binding from undifferentiated prosensory progenitors in the auditory sensory epithelium at E13.5 to differentiation at E16.5 (Figure 1A). To better characterize the chromatin structure associated with Six1, we also used antibody-mediated ChIP on E13.5 cochleae to pull-down chromatin associated with the histone H3 Lys 27 acetylation (H3K27ac)—an epigenetic marker associated with active enhancers (26) and the histone H3 Lys 27 trimethylation mark (H3K27me3)—an epigenetic marker associated with transcriptional repression (27). The peak bed files were generated from peak calling against both genomic input DNA and IgG ChIP-seq controls with the default setting and the resulting common peaks from these two bed files were used for subsequent analyses. We identified a total of 14 967 Six1-bound regions and observed clusters with varying levels of enrichment (Figure 1B and Supplementary Figure S1A). 5270 regions showed loss of or reduced Six1-occupancy at E16.5 with very weak or no H3K27ac-deposition (cluster I, ‘precursor-transient peaks’), while 6616 regions (cluster II, ‘differentiation peaks’) showed new or increased binding at E16.5 with weaker H3K27ac-deposition at E13.5. 2981 Six1-bound sites retained occupancy upon differentiation and had strong H3K27ac-deposition (cluster III, ‘persistent peaks’), indicating that these regions are enhancers from E13.5. ∼66% of E16.5 and 37% of E13.5 peaks were marked by H3K27ac (Figure 1C and Supplementary Figure S1D, E), suggesting an increase in the proportion of Six1-bound enhancers as differentiation proceeds. We also identified a total of 7558 genes associated with these Six1 peaks by assigning peaks to their single nearest genes within 500 kb of the nearest gene's TSS (transcription start site) using GREAT analysis (Figure 1C and Supplementary Files S1–S3). Among them, 3214 were common to both stages and 1551 or 2793 were E13.5- or E16.5-specific genes respectively (Figure 1C). The three clusters of peaks share common genes due to multiple distinct peaks per gene.

We also observed stage-specific differences in the genomic distribution of Six1 peaks. The majority (∼96%) of E13.5 peaks were intronic and intergenic (Figure 1D) and 84% of the precursor-transient peaks were distal regions >5 kb from TSSs (transcriptional start sites) of the nearest genes (Supplementary Figure S1C). By E16.5, the number of intronic peaks remained similar, but the proportion of intergenic Six1 sites was drastically reduced, while the proportion of promoter–TSSs sites was increased (Figure 1D, E and Supplementary Figure S1C). Thus, this analysis reveals the highly dynamic nature of Six1-binding patterns during cell fate induction and subsequent differentiation in the auditory sensory epithelium development. The higher density of E16.5 peaks in the vicinity of the TSSs likely reflects the functional relevance of these sites in regulating differentiation genes.

### Six1 binds to a broad set of key loci to drive sensory epithelium formation

GREAT and Gene Ontology analyses revealed overrepresentation of genes related to inner-ear/cochlea development in Six1 targets (Figure 1F and Supplementary Figure S1B). Notably, differentiation peaks were significantly enriched for terms related to molecular function of voltage-gated chloride channel activity, actin filament binding, and single-stranded RNA binding (Supplementary Figure S1B).

The global analyses indicated that Six1-occupancy to putative CREs is dynamic over the time. To illustrate this behavior, we highlighted Six1’s associations with several targets that are active in E13.5 cochlea (without deposition of the repressive mark H3K27me3) and involved in the Wnt, Notch, Shh, and Fgf signaling pathways that are crucial for prosensory primordium specification and both hair cell and supporting cell fate selection. The Wnt signaling mediator Lgr5 is expressed in prosensory progenitors and maintained in a subset of supporting cells during differentiation (28). Recent studies found that Lgr5^+^ cells are capable of differentiating into hair cells in response to Wnt signaling (29). We identified three Six1-bound regions with H3K27ac-deposition at the *Lgr5*: two persistent (one promoter-proximal and one distal intronic region ∼+66-kb) and one precursor-transient intronic region ∼+94-kb (Figure 1G), which were confirmed by ChIP-qPCR (Figure 1H). Similar dynamics were observed in Six1 peaks at *Wnt5a/Tcfs*, and in the Notch (*Notch1,2/Jag1/Rbpj/Hes1,5/Hey1*) (Figure 1G,H and Supplementary Figure S2), Shh (*Gli3*/*Mycn*/*Tulp3*), BMP (Bmp2,3,4,5,6,7,8/Bmper/Bmpr1a,1b), and Fgf (*Fgfr1,2,3/Fgf1,7,8,9,10,16,17,18,20,21* and *Dusp1,4,6,7,10,11,14,16,18,26*) (Supplementary Figure S2) pathways. Stage-associated changes in Six1 peaks were also observed at loci encoding TFs that are essential for sensory epithelium development and cell fate induction, including *Six1* itself, *Sox2/4*, *Pax2, Hes1* and *Hey1* (Figure 1G and Supplementary Figure S2).

In summary, our Six1 ChIP-seq demonstrates the time course of binding dynamics of this key TF in both hair and supporting cell fate selection and subsequent differentiation, thus uncovering a broad role for Six1 in auditory sensory epithelium development.

### Six1 occupies enhancer repertoire to regulate sequential induction of key TFs that then engage in protein complexes

**Figure 2.**
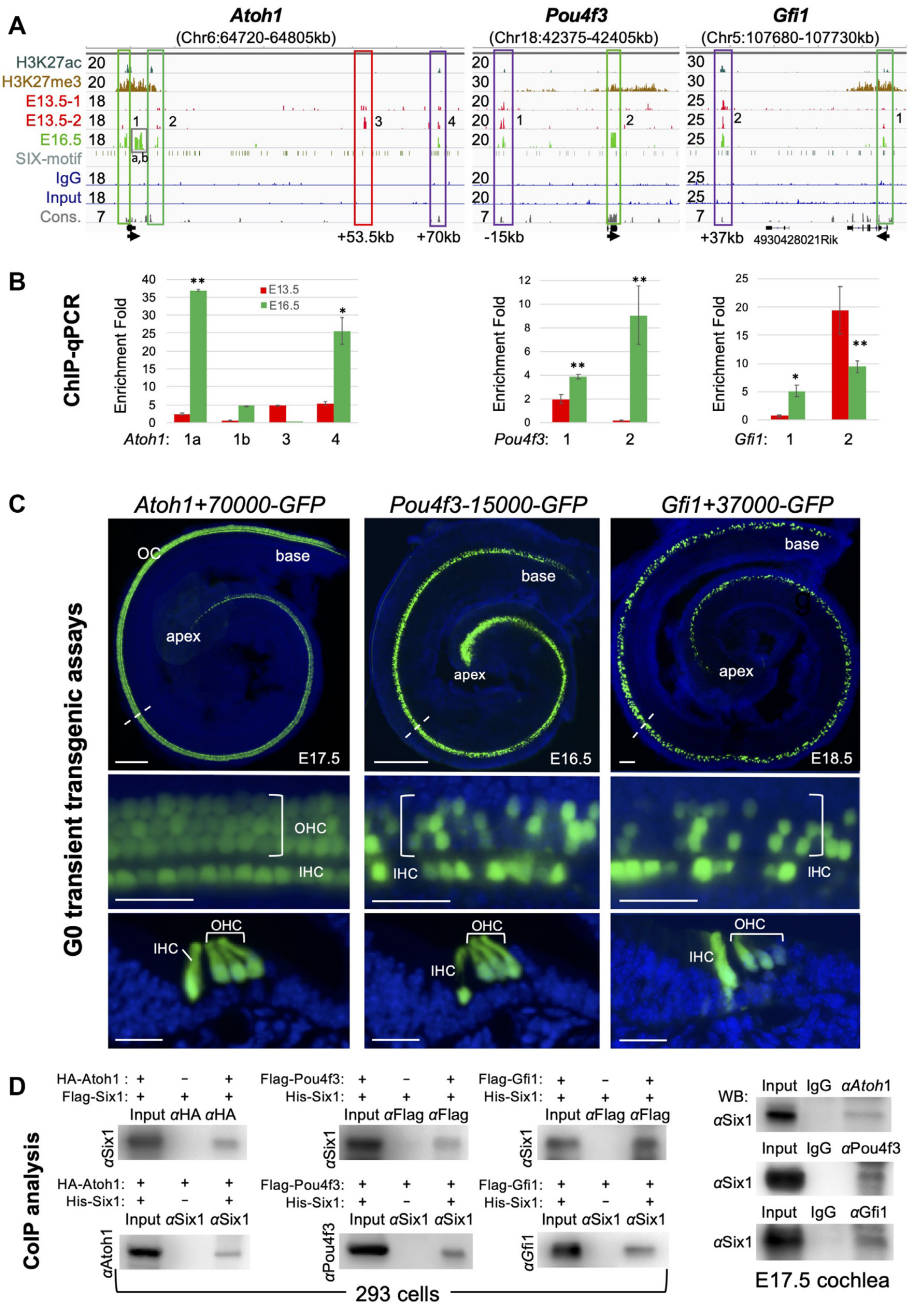
Six1 occupies enhancer repertoire to induce sequential activation of Atoh1, Pou4f3 and Gfi1 that then engage in protein complexes. (**A**) Genome browser visualization of Six1 peaks at *Atoh1, Pou4f3* and *Gfi1*. Note that the proximal peak-2 of *Pou4f3* is in exon. y-Axis numerical values in each track indicate track height scaling in read depth. Sequence conservation (cons.) is indicated. The arrow at the TSS points to the direction of transcription. (**B**) ChIP-qPCR analysis of the boxed peaks in (A). IPs and mock IPs were normalized to inputs and the enrichment of mock IP was considered 1-fold (not shown). *P < 0.05, **P < 0.01. (**C**) Transient (G0) transgenic analysis of a 552-bp Six1-bound *Atoh1+70000* (in 5/5 transgenic lines), *Pou4f3-15000* (in 3/3 transgenic lines) or *Gfi1+37000* (in 3/3 transgenic lines) driving GFP reporter showing the HC-restricted activity of these distal enhancers. Top panels, whole-cochlea images; middle panels, higher magnification of the areas indicated by dashed lines; lower panels, images of cochlear sections showing GFP^+^ hair cells in the organ of Corti. Scale bars: 100 μm for top panels and 30 μm for middle and bottom panels. (**D**) Co-immunoprecipitation (coIP) analysis of nuclear extracts from E17.5 cochleae or 293 cells cotransfected with indicated plasmids. Antibodies used for IP or for western detection are indicated. Anti-HA or -Flag was used for immunoprecipitating HA-Atoh1 or Flag-Pou4f3/Flag-Gfi1 fusion protein.

The hair cell fate is induced upon activation of Atoh1 (30–32), which regulates the expression of downstream TFs Pou4f3 (33) and Gfi1 (34). Before the onset of hair cell differentiation ∼E14.5, all three genes had H3K27me3-deposition at E13.5. We previously identified *Atoh1* as a target of Six1 based on the dual criteria of changes in *Atoh1* expression in response to *Six1* loss- or gain-of-function experiments and Six1-binding to the 1.4-kb 3′-*Atoh1* autoregulatory enhancer (14,16,35). ChIP-seq revealed Six1-binding to this region (peak-2, increased by E16.5, Figure 2A). Six1-occupancy to a promoter-differentiation peak (peak-1a,b) was also identified. This region has been previously reported as a target of the Notch mediator Hes/Hey repressor families for supporting cell fate selection (36). Notably, two distal regions—a precursor-transient peak-3 ∼+53.5-kb and a persistent/differentiation peak-4 ∼+70-kb – were also occupied by Six1. ChIP-qPCR confirmed stage-related changes in Six1-occupancy and revealed a significant increase in Six1-binding to peak-1a and peak-4 by E16.5 (Figure 2B).

To further examine the functional roles of the Six1-bound *Atoh1* CREs, we examined activity of the two novel distal regions using mouse transient transgenic assays. The precursor-transient 500-bp of *Atoh1*+53 500 had no activity in E17.5–E18.5 cochlea (3/3 transgenic lines, data not shown), suggesting that this precursor Six1-occupancy may ‘prime’ *Atoh1* by limiting binding to other TFs. In contrast, a 500-bp of *Atoh1*+70 000 drove hair-cell-restricted expression in all inner-ear sensory organs in all five transgenic lines (Figure 2C) with 3/5 lines showing a mosaic expression pattern (Supplementary Figure S3A), which often occurs in pronuclear injection where DNA is integrated in a two-cell or later stage embryo. Thus, we have identified a novel Six1-bound distal *Atoh1* enhancer and have found that Six1 targets both proximal and distal CREs to regulate *Atoh1* expression ‘in time and space’ to specify hair cell fate.

At the *Pou4f3* locus, a distal persistent peak-1 ∼–15-kb was identified and confirmed by ChIP-qPCR with stronger enrichment at E16.5 than at E13.5 (Figure 2B). *Gfi1* is a target of *Pou4f3* (34) and at the *Gfi1* locus, a persistent peak ∼+37-kb and a differentiation peak near the promoter-TSS were identified (Figure 2A). In transgenic assays, both *Pou4f3*-15 000 and *Gfi1*+37 000 drove HC-restricted expression in all sensory organs (Figure 2C and Supplementary Figure S3B, C). Multiple peaks were also identified at *Gata3* (Supplementary Figure S3D), which was reported to synergize with *Atoh1/Pou4f3* to convert supporting cells to hair cells in young mice (37). Co-immunoprecipitation (coIP) revealed complex formation of Six1 with Atoh1, Pou4f3, Gfi1 or Gata3 in cochlea or 293 cells (Figure 2D and Supplementary Figure S3E). Together, these data indicate that Six1 acts in a positive feedforward loop in which it regulates Atoh1, which then forms protein complexes to autoregulate Atoh1 to increase its expression from E13.5 to E17.5 (30) and regulate the expression of downstream TFs Pou4f3 or Gfi1 that then cooperatively control targets through direct binding to CREs/enhancers in order to drive the precise timing of hair cell fate specification and stepwise differentiation.

Intriguingly, we discovered that Six1 pre-occupies CREs of hair-cell-subtype-specific genes at the precursor stage, including inner-hair-cell-specific *Calb2* (Calretinin) and outer-hair-cell-specific *Slc26a5* (Prestin) (Supplementary Figure S3F). Six1-occupancy was also observed in supporting-cell-subtype-specific genes, including *S100a* (inner-hair, inner-phalangeal/Deiters' cells) and *Slc1a3* (GLAST, inner-phalangeal/inner-border cells). Thus, Six1 may engage target sites in chromatin for later activation.

### Six1 binds DNA at sites carrying consensus sequences for CTCF/BORIS and RFX

**Figure 3.**
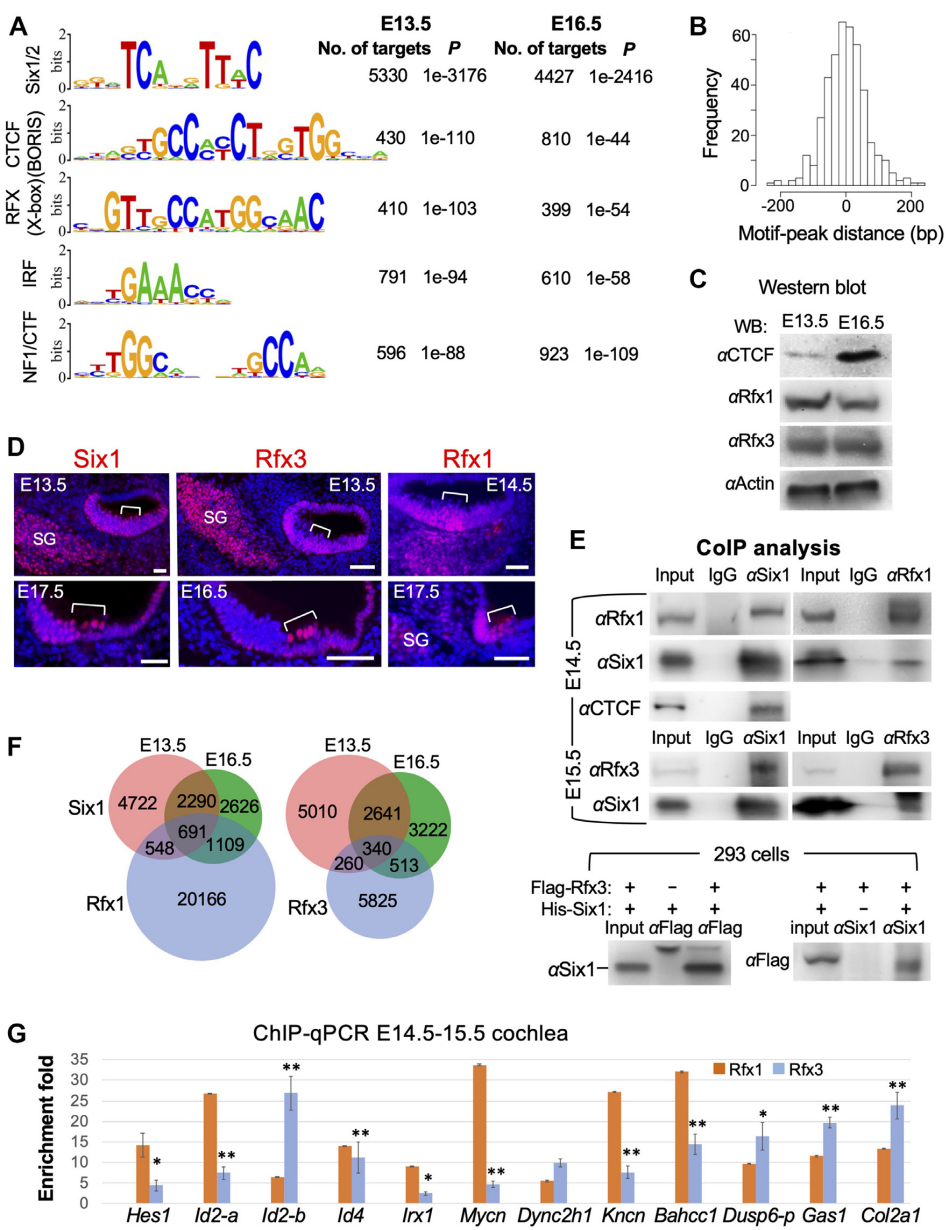
Motif analysis of Six1 peaks and physical interaction with RFX and CTCF. (**A**) Sequence logos of the most enriched top 5 motifs from Homer Known motif analysis. (**B**) Localization of Six1/2-motif within the peak sequence. (**C**) Western blot analysis of whole-cochlea extracts with indicated antibodies. (**D**) Immunostaining on cochlear sections showing Six1, Rfx3 and Rfx1 expression in the organ of Corti (brackets). Scale bars: 45 μm. (**E**) CoIP analysis using nuclear extracts from E14.5–15.5 cochleae or 293 cells cotransfected with *Flag-Rfx3* and *His-Six1* plasmids (*Rfx1* expression plasmid is unavailable). Anti-Flag is used for precipitating and detecting Flag-Rfx3 fusion protein expressed in 293 cells. (**F**) Venn diagram indicating overlap of Six1-binding sites with Rfx1- or Rfx3-bound sites in the mouse Min6 cells (42). (**G**) ChIP-qPCR of 12 selected common peaks for Six1 and Rfx1/3 confirms binding of Rfx1 and/or Rfx3 to these regions. IPs and mock IPs were normalized to inputs and the enrichment of mock IP was considered 1-fold (not shown). *P < 0.05, **P < 0.01.

As expected from a direct association of Six1-DNA, the most enriched motif (*P* = 10^−3138^ or *P* = 10^−2311^) matched to the Six1/2-binding motifs (Figure 3A), the majority of which were enriched at the peak center within ±200-bp (Figure 3B). A higher proportion of peaks at E16.5 (∼54%) than E13.5 (∼35%) lacked Six1/2-binding sites, suggesting an indirect association of Six1 to DNA through interactions with DNA-binding proteins.

Examining the presence of known motifs revealed that CTCF/BORIS, RFX/X-box (HTH), IRF and NF1/CTF are among the top five most enriched motifs (Figure 3A). CTCF/BORIS are essential epigenetic components with a primary role in the organization of global chromatin architecture (38). CTCF has a role in auditory sensory epithelium development but not in HC formation (39,40). The NF1/CTF (CAAT box-binding/nuclear factor-1) is a widely expressed TF that controls DNA transcription and replication (41). While it is unclear if IRF proteins have a role in the inner ear, the RFX proteins Rfx1/3 were recently reported to have a redundant role in differentiating hair cells at postnatal stage (42). Other highly overrepresented motifs include SOX, bHLH, homeobox, and TCF proteins that are known to interact with the SIX family proteins (14–15,43) (Supplementary Figure S4A). Interestingly, novel motifs for ETS, Tlx (NR), Forkhead and TEAD proteins were also significantly enriched (Supplementary Figure S4A). Consistent with the coIP analyses, additional motifs for Atoh1, Gfi1, OCT/POU and GATA were enriched to a lesser degree (Supplementary Figure S4A). This analysis provides insight that potential TFs with these combinatory motifs may act as critical components of Six1-bound CREs functioning in vivo.

We next focused on examining if Rfx1/3 collaborate in Six1-DNA interactions due to their importance in differentiated hair cells. Western blot and immunohistochemistry confirmed the expression of Rfx1/3 in the sensory epithelium of E13.5–16.5 cochlea (Figure 3C, D). CoIP analysis revealed complex formation between Six1 and Rfx1/3 or CTCF in cochlea or 293 cells (Figure 3E). Comparison of Six1 ChIP-seq data with published Rfx1/3 ChIP-seq in mouse Min6 cells (42) showed 2348 or 1113 of Six1 peaks co-occupied by Rfx1 or Rfx3 respectively (above 70% of them are <5 kb to TSS) (Figure 3F). We selected 12 common peaks and performed ChIP-qPCR using chromatin from E14.5–E15.5 cochleae to confirm in vivo occupancy of Rfx1 or Rfx3 for all 12 regions (Figure 3G). As Six1 also occupies proximal–promoter of *Rfx1* and *Rfx3* (Supplementary Figure S4B), Six1 may act in a similar positive feedforward loop to form protein complexes with RFX to synergistically coregulate their targets during differentiation.

### Dependence of enhancer activity on co-binding of Six1-Rfx1/3

**Figure 4.**
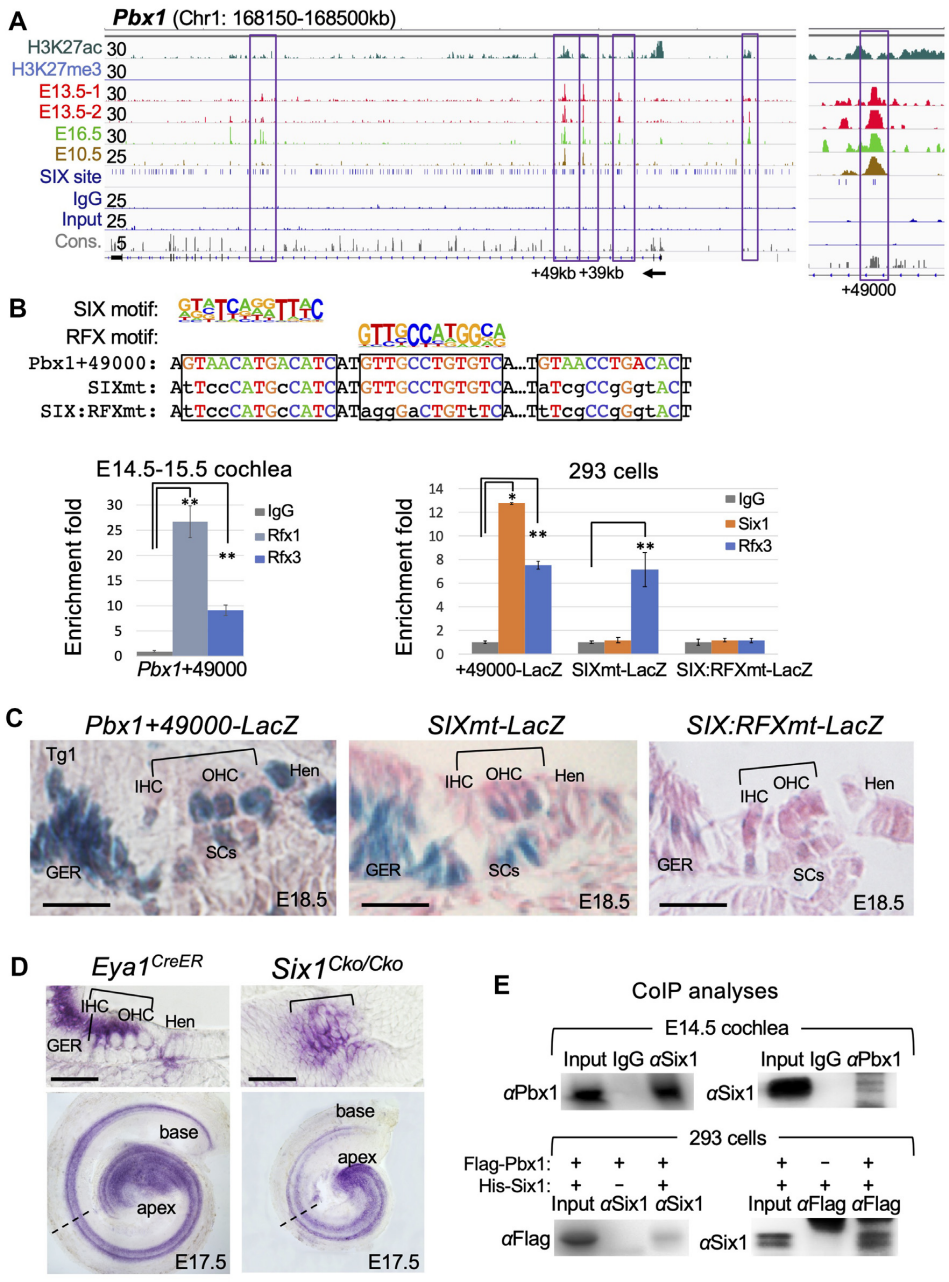
Dependence of a distal *Pbx1* enhancer activity on co-binding of Six1 and Rfx1/3 through SIX:RFX motifs. (**A**) Genomic browser visualization of multiple Six1-bound regions at the *Pbx1* and enlarged view of the intronic peak at ∼49-kb downstream from the TSS. (**B**) *Pbx1*+49 000 contains two SIX-motifs separated by a RFX-motif. ChIP-qPCR using chromatin from E14.5–E15.5 cochlear epithelium shows strong binding with Rfx1 and relative weaker binding with Rfx3. A 510-bp fragment of Pbx1+49000 driving *LacZ* reporter transgene and two mutant reporter transgenes were generated by introducing mutations into the predicted SIX-motifs or both SIX:RFX-motifs in combination. These reporters were assessed by ChIP-qPCR using chromatin prepared from 293 cells cotransfected with His-Six1 expression plasmid and reporter *Pbx1*+49 000, *Pbx1*+4900SIXmt or *Pbx1*+49000SIX:RFXmt. These mutations abolished Six1 or Rfx3 binding. Transfection was repeated three times and qPCR was performed in triplicates for each independent experiment. Input was used for normalization (see Materials and Methods) and the enrichment of mock IP was considered 1-fold. *P < 0.05, **P < 0.01. (**C**) G0 transgenic analysis of *LacZ* transgene driven by a 510-bp of *Pbx1*+49 000 showing activity in the sensory epithelium and flanking GER and Hensen's (Hen) ells (*n* = 7/7 transgenic embryos), while *Pbx1*+49 000SIXmt (*n* = 3/3 transgenic embryos) or *Pbx1*+49000SIX:RFXmt (*n* = 8/8 transgenic embryos). Brackets indicate the organ of Corti. (**D**) *In situ* hybridization of E17.5 *Eya1^CreER^* or *Six1^Cko/Cko^* (*Eya1^CreER^;Six1^fl/fl^*, tamoxifen given at E12.5). Top panels, sections of whole-cochlea shown in bottom panels indicated by dashed lines. Brackets indicate the organ of Corti. (**B**) Genomic browser visualization of multiple Six1-bound regions at the *Pbx1* and enlarged view of the intronic peak at ∼49-kb downstream from the TSS. (**E**) CoIP analysis of nuclear extracts from E14.5 cochleae or 293 cells transfected with *Flag-Pbx1/His-Six1*. Other abb.: GER, greater epithelial ridge; IHC, inner hair cell; Hen, Hensen's cells; OHC, outer hair cell; SCs, supporting cells. GER, greater epithelial ridge. Scale bars: 30 μm.

To investigate whether Six1-RFX coregulate targets through common CREs, we selected *Pbx1* due to the presence of multiple Six1-bound regions at this gene and its unknown function in the inner ear. Six1 occupies two distal regions ∼+39-kb and ∼+49-kb at E10.5 (Figure 4A) and *Pbx1*+49000 with higher sequence conservation contains two SIX-motifs separated by an RFX-motif (Figure 4B). ChIP-qPCR confirmed Rfx1/3-binding to this region in both cochlea and 293 cells cotransfected with a reporter transgene driven by a 510-bp of *Pbx1*+49000 and Six1 or Rfx3 expression plasmid respectively (Figure 4B). A 4-bp mutation of each of the predicted SIX-motifs and a 5-bp mutation of the RFX-motif abolished Six1- or Rfx3-binding (Figure 4B).

In transgenic embryos, the 510-bp of *Pbx1*+49 000 was active in the otocyst (Supplementary Figure S4D, E), cochlear hair cells and flanking nonsensory cells (*n* = 7/7 transgenic lines, Figure 4C), recapturing the pattern of *Pbx1* mRNA expression detected by in situ hybridization (Figure 4D and Supplementary Figure S4C). However, β-Gal activity was also found in supporting cells in the sensory epithelium (Figure 4C), which is likely due to lack of cooperative interactions with repressive elements that are present in the locus. The 4-bp mutation of SIX-binding sites did not completely disrupt the activity in the otocyst (Supplementary Figure S4F), but did decrease activity in the auditory hair cells (*n* = 3/3 transgenic lines, Figure 4C). However, mutation of both SIX:RFX motifs disrupted enhancer activity in the otocyst (Supplementary Figure S4G) and cochlear epithelium, including the flanking nonsensory GER (greater epithelial ridge) and Hensen's cells (*n* = 8/8 transgenic lines, Figure 4C), and some β-Gal activity was only observed in an ectopic region above the GER toward the roof of the cochlear duct (arrow, Supplementary Figure S4J). Similar observation was obtained from vestibular sensory organs (Supplementary Figure S4H,I). These results suggest that Six1 and RFX proteins act synergistically to coregulate the expression of *Pbx1* via direct binding to the SIX:RFX motifs of *Pbx1*+49 000 enhancer.

Consistent with the decreased transgene activity in the cochlear epithelium driven by the SIXmt enhancer, examination of *Pbx1* mRNA expression in *Six1^Cko/Cko^* cochlea revealed decreased *Pbx1* expression in hair cells, GER and Hensen's cells in *Six1*-deficient cochlea (tamoxifen given from E12.5 using *Eya1^CreER^*) compared to control littermates (Figure 4D). This further confirms that *Pbx1* expression in the cochlea is partly dependent on Six1 activity.

CoIP analysis found that Pbx1 and Six1 also form protein complexes both in vivo and in vitro (Figure 4E), which is consistent with the identification of Pbx1-motif in Six1 peaks (Supplementary Figure S4A). Altogether, these results identify Pbx1 as both a functional target and a novel partner TF of Six1, acting in a similar positive feedforward regulation of sensory epithelium development.

### Six1 regulates the expression of *Fgf8* and effector *Dusp6* of the Fgf signaling in the sensory epithelium through directly binding to cell-subtype-specific enhancers

**Figure 5.**
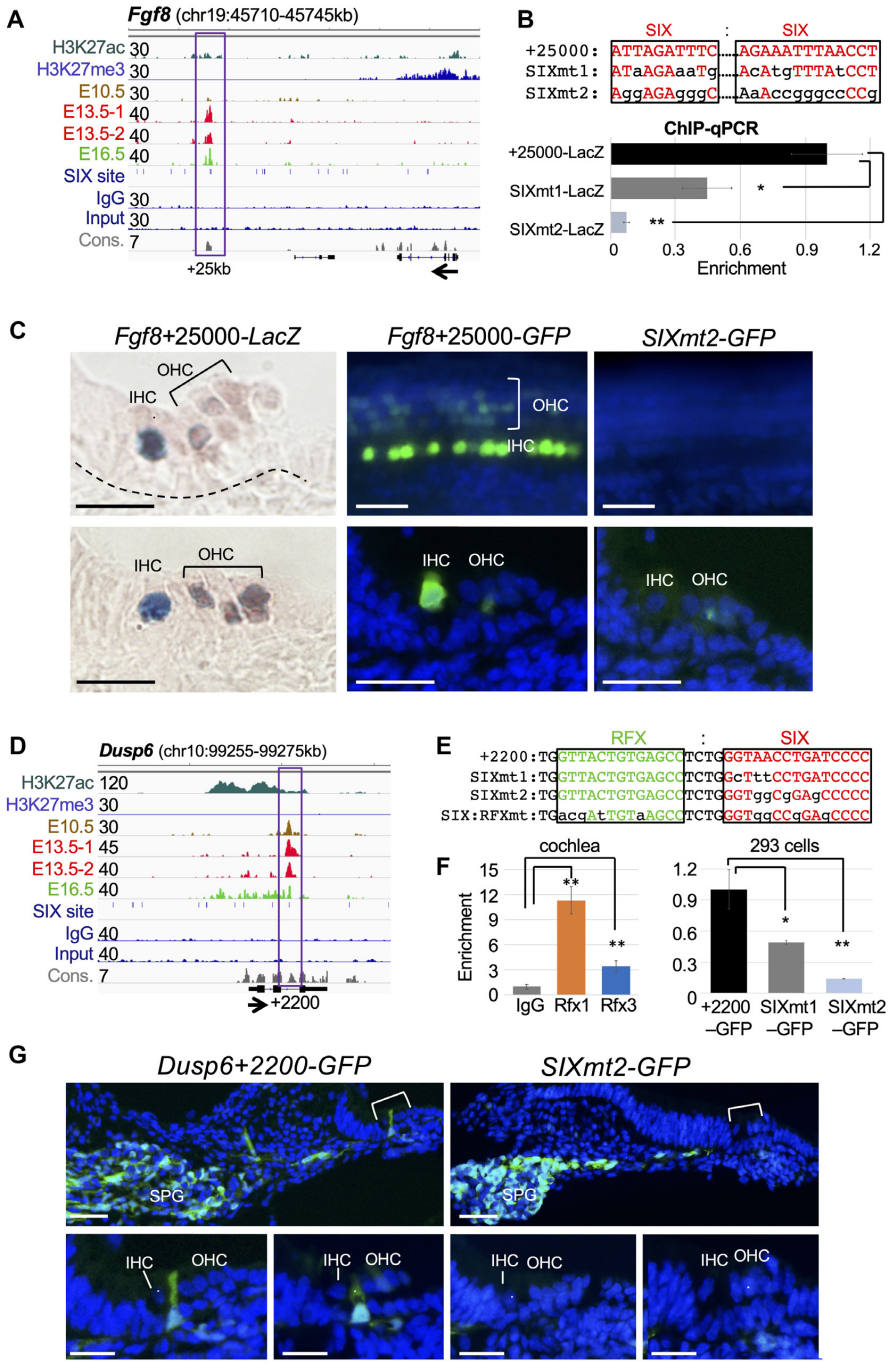
Six1 directly regulates cell-type-specific genes/effectors of the Fgf signaling. (**A**) Genome browser visualization of Six1 peak ∼25 kb downstream from the TSS of *Fgf8*. Note: no enrichment of Six1 in E10.5 otocyst. (**B**) Two mutant enhancers SIXmt1 and SIXmt2 driving *LacZ* or *GFP* reporter were generated respectively by introducing different mutations into the Six1/2-motifs and assessed by ChIP-qPCR using chromatin prepared from 293 cells cotransfected with His-Six1 expression plasmid and reporter *Fgf*8+25000, *Fgf8*+25000SIXmt1 or *Fgf8*+25000SIXmt2. **P < 0.01 and ***P < 0.001. (**C**) G0 transgenic analysis of *LacZ* or *GFP* transgene driven by *Fgf*8+25000 or *Fgf8+25000SIXmt2*. Left panels, images of two cochlear sections showing strong activity in inner hair cells and very weak in outer hair cells (*n* = 9/9 transgenic embryos), which was disrupted by *SIXmt2* (*n* = 7/7 transgenic embryos). Middle and left upper panels, images of whole-cochlea showing the organ of Corti; middle and right bottom panels, images of sections of upper panels. (**D**) Genome browser visualization of intronic Six1 peak ∼2200 bp downstream of the TSS of *Dusp6*, which contains RFX-motif next to the SIX motif with high conservation (con.). (**E**) Sequences indicate distinct mutations introduced into the SIX motif or both SIX:RFX motifs. (**F**) ChIP-qPCR analysis of chromatin prepared from E14.5–E15.5 cochleae showing stronger enrichment for Rfx1 and relatively weaker enrichment for Rfx3 at *Dusp6*+2200, while ChIP-qPCR of chromatin prepared from 293 cells cotransfected with His-Six1 expression plasmid and reporter driven by *Dusp6+2200*, *Dusp6+2200SIXmt1* or *SIXmt2*. **P < 0.01. (**G**) G0 transgenic analysis of the *Dusp6*+2200 or *Dusp6*+2200SIXmt2. Images of cochlear sections showing GFP transgene expression in spiral ganglion (SPG) and inner-pillar-cells in the sensory epithelium at E18.5 (*n* = 8/8 transgenic embryos). SIXmt2 disrupted enhance activity specifically in the inner-pillar-cells in the sensory epithelium (*n* = 5/6 transgenic embryos). Scale bars: 20 μm.

We next characterized the activity of Six1-bound CREs in Fgf signaling, which plays diverse roles in auditory sensory epithelium formation and growth (44). We previously identified *Fgf8* as a target of Six1 based on its decreased expression in *Six1*-deficient inner hair cells (16). Six1 ChIP-seq identified two distal-persistent peaks ∼+25-kb and ∼+67-kb and a proximal-differentiation peak ∼–4.5-kb at *Fgf8* (Supplementary Figure S2). Examination of *Fgf8+*25000 (Figure 5A) *in vivo* showed strong activity restricted to *Fgf8*-expressing inner hair cells (*n* = 9/9 transgenic lines, Figure 5C). Expansion of weak activity in outer hair cells is likely due to lack of cooperative interactions with repressive elements in the locus. This region contained two Six1/2-motifs separated by a GATA and a bHLH-binding E-box motifs. We generated a LacZ or GFP reporter transgene driven by a 714-bp fragment of *Fgf8*+25000 and introduced two mutations of the predicted SIX-motifs (SIXmt1 and SIXmt2) (Figure 5B). ChIP-qPCR using chromatin from 293 cells cotransfected with *Six1* expression plasmid and the *Fgf8*+25 000, SIXmt1 or SIXmt2 reporter transgene found that SIXmt1 only decreased Six1-binding in 293 cells (Figure 5B) and weakened enhancer activity *in vivo* (*n* = 5/5 transgenic lines), whereas SIXmt2 disrupted Six1-binding in 293 cells (Figure 5B) and abolished transcriptional activity *in vivo* (*n* = 7/7 transgenic lines, Figure 5C). This demonstrates that Six1-binding is necessary for inner hair cell-specific enhancer activity.

*Dusp6* is a downstream effector of Fgfr signaling and inactivation of *Dusp6* causes hearing loss (45,46). *In vivo* examination of *Dusp6*+2260 with strong H3K27ac-deposition (Figure 5D) revealed activity in the otocyst, cochlear inner-pillar cells and the spiral ganglion (*n* = 8/8 transgenic lines, Supplementary Figure S5B, C and Figure 5G), recapitulating the pattern of *Dusp6* expression (47) (Supplementary Figure S5A, D). This region contains an RFX-motif adjacent to the SIX-motif (Figure 5E) and ChIP-qPCR on E14.5–E15.5 cochleae confirmed stronger enrichment by Rfx1 than Rfx3 (Figure 5F). A 3-bp mutation of the SIX-motif (SIXmt1) reduced Six1-binding (Figure 5E, F) and weakened enhancer activity (*n* = 3/3 transgenic lines). However, a 4-bp mutation of SIX alone (SIXmt2), which completely disrupted Six1-binding (Figure 5E, F), or mutation of both RFX:SIX motifs abolished enhancer activity in the sensory epithelium, while spiral ganglion activity remained unperturbed (*n* = 5/6 transgenic lines, Figure 5G). Although the RFX-motif is non-redundant for enhancer activity *in vivo*, co-binding with Rfx1/3-binding may affect Six1-DNA binding affinity.

In contrast to the presence of *Pbx1* expression in *Six1*-deficient cochlea, *Dusp6* expression was almost completely lost in *Six1*-deficent cochlear sensory epithelium with residual expression in the apical end (Supplementary Figure S5D). This indicates that *Dusp6* expression *in vivo* requires Six1 activity. Collectively, these data indicate that Six1 directly regulates inner-pillar-cell-specific *Dusp6* expression by binding to the intronic *Dusp6*+2200 enhancer.

### Inactivation of *Six1* in differentiating hair cells disrupts both hair-bundle structural polarity and planar cell polarity (PCP)

**Figure 6.**
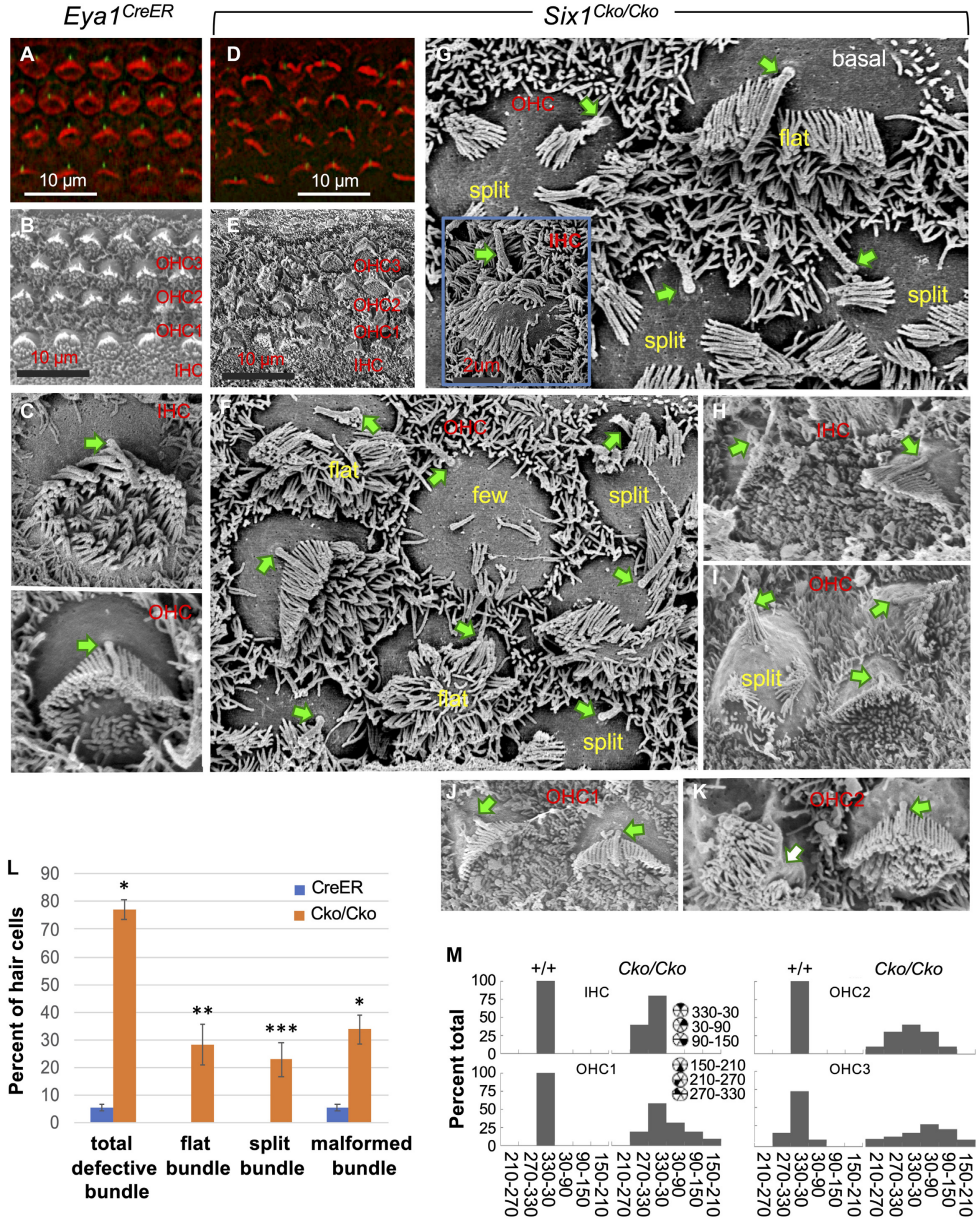
Late conditional inactivation of *Six1* in differentiating hair cells results in both structural polarity and PCP defects of hair-bundles. (**A**, **D**) Hair-bindle structure and orientation at P0 was visualized by F-actin (red) and anti-acetylated tubulin (green, for kinocilium). (**B**–**K**) SEM images from basal or middle cochlear duct showing surface views of the organ of Corti in *Eya1^CreER^* control and *Six1^Cko/Cko^* mutant. Arrows indicate kinocilium (C–J), which is absent in panel K. (**L**) Percentage of hair cells from the basal region of the cochlea of control (*n* = 645; 3 embryos) and Rac1 and CKO (*n* = 622; 3 embryos) with the indicated stereocilia. *P < 0.05, ** *P* < 0.01, *** *P* < 0.001. (**M**) Graphs showing distribution of hair cell orientation from wild-type and *Six1* CKO mutant animals. The orientation of hair cells was determined by measuring the angle formed between the medial-to-lateral axis of the cochlea and the line bisecting the stereociliary bundle from the center of the hair cell to the vertex of the hair-bundle.

It is currently unknown whether and how this key TF regulates hair-bundle morphogenesis during terminal differentiation. To bridge our ChIP-seq data to cellular differentiation of the auditory sensory epithelium, we conditionally deleted *Six1* in differentiating hair cells (tamoxifen at E14.5). On the apical surface, F-actin and anti-acetylated tubulin staining and scanning electron microscopy (SEM) revealed V-shaped stereocilia packed with actin filaments and a kinocilium centered next to the tallest stereocilia on each hair cell, which are uniformly aligned along the medial-lateral axis across the entire sensory epithelium (referred as PCP) (Figure 6A, B). The stereocilia and kinocilium are interconnected by distinct types of hair-bundle links to maintain the intrinsic structural polarity. SEM also revealed flatter inner hair cell bundles and V-shaped outer hair cell bundles at P0 (Figure 6C). The apical surface of *Six1^Cko/Cko^* sensory epithelium displayed disrupted intrinsic polarity and PCP with a range of both structural deformation and misorientation (Figure 6D-K).

The primary hair-bundle defects include flat bundle (Figure 6F, G), multiple groups of stereocilia within the same cell (split) (Figure 6F, G, I), and very few stereocilia (Figure 6F). The kinocilium was present on the lateral edge of the hair cell apical surface, indicating that kinocilia normally migrate from the center. However, the kinocilia were often found off-centered without connection to the stereocilia (Figure 6F, G). Occasionally the kinocilium was found either centered within one group of stereocilia (Figure 6I) or absent (white arrow, Figure 6K). Overall, 78% of hair cells counted from the mid-basal cochlea displayed hair-bundle abnormalities (Figure 6L).

The bundle orientation as a readout of hair cell PCP was also significantly disrupted in both inner hair cells and outer hair cells with outer hair cells more affected than inner hair cells (Figure 6F–K). The angle measurements of misoriented bundles varied with some bundles in outer hair cells rotated up to 90–150° (Figure 6K, M). Together, these observations demonstrate the importance of Six1 in both cell-intrinsic bundle morphogenesis and PCP during terminal differentiation.

### Six1 targets a wide range of regulators involved in development of primary hair-bundle and orientation

**Figure 7.**
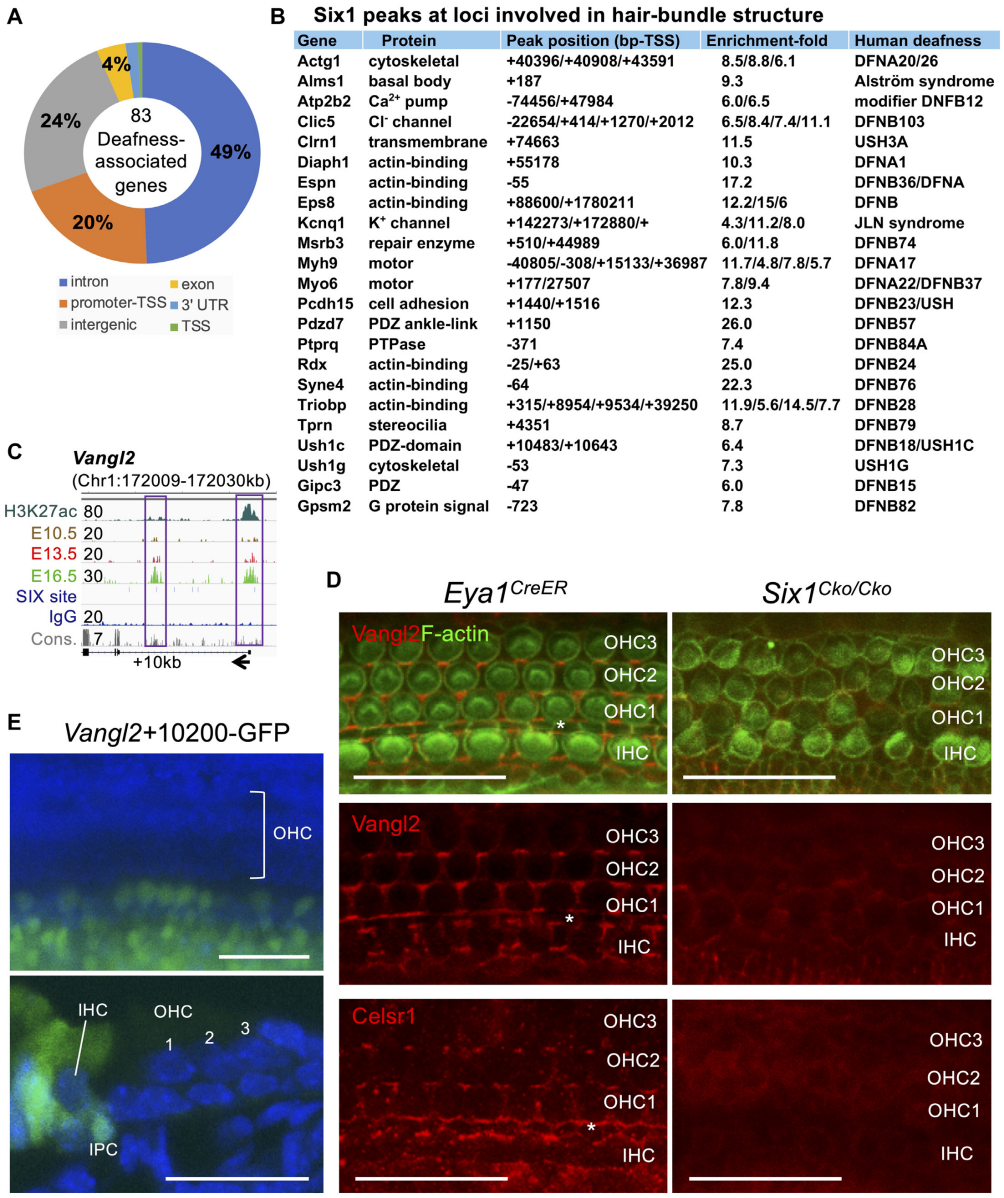
Six1 occupies key loci that are responsible for different forms of human deafness syndromes and for development of primary hair-bundle and orientation. (**A**) Genomic distribution of the 186 Six1 peaks that are mapped to 83 of the 152 deafness-associated genes collected in the Deafness Variation Database using UCSC liftOver. (**B**). List of Six1-enrichments and peak locations in the mouse genome for 23 homologs of the 83 deafness-causing genes. For a complete list of peak locations in the 83 human genes, see Supplementary file 4. (**C**). Genome browser visualization of Six1 peak at proximal-promoter and intronic regions of *Vangl2*. Six1-binding to both regions increases by E16.5. (**D**). Surface views of the organ of Corti stained with both anti-Vangl2 and F-actin or anti-Celsr1 alone. Asterisk indicates the position of pillar-cells between inner and outer hair cells. (**E**). G0 transgenic analysis of a 552-bp of the intronic *Vangl2*+10200 showing enhancer activity in inner hair cell and surrounding SCs on the medial region of the sensory epithelium as well as in the GER (in 3/4 transgenic embryos/lines). IPC, inner-pillar-cell. Scale bars: 30 μm.

Using UCSC liftOver (48), we mapped Six1 peaks to a total of 7495 genes in the human genome and found that 186 peaks mapped to 83 of the 152 deafness-associated genes collected in the Deafness Variation Database (49) (Figure 7A and Supplementary file S4), which overlapped with over 2101 SNPs, of which >80% belong to unknown significance (Supplementary Figure S6A). Notably, mutations in many of these genes cause deafness due to hair-bundle abnormalities (50). These targets include myosin motors, actin binding, cytoskeletal, scaffolding, transmembrane, cell adhesion, multiple channel proteins, and G-protein signaling (Figure 7B). Prominent Six1 targets of the PCP-signaling include *Vangl1/Vangl2/Celsr1* and *Ptk7* (Figure 7C and Supplementary Figure S6B). Immunostaining for Clic5, which is localized to hair-bundle (51), revealed significant reduction in *Six1^Cko/Cko^* (Supplementary Figure S7A). As indicated by F-actin and anti-Vangl2 or -Celsr1 staining (Figure 7D), while disorganization of cell-to-cell contacts was apparent in *Six1^Cko/Cko^*, the expression of two key components of the Wnt/PCP signaling Vangl2/Celsr1, which are localized on the medial side of the hair cell membranes (52,53), was markedly reduced in the CKO sensory epithelium. qRT-PCR confirmed decreased expression of these targets in *Six1^Cko/Cko^* (Supplementary Figure S7B).

Surprisingly, in vivo examination of the intronic *Vangl2*+10200 CRE (Figure 7C) found activity in inner hair cells and surrounding supporting cells on the medial region of the sensory epithelium as well as in GER, but not in outer hair cells and their surrounding supporting cells on the lateral sensory epithelium (Figure 7E and Supplementary Figure S7C). This CRE was also active in all vestibular hair cells and supporting cells (Supplementary Figure S7C). Thus, *Vangl2* expression in medial versus lateral auditory sensory epithelium is mediated through distinct CREs. Collectively, these results provide insight into how Six1 regulates terminal differentiation through direct binding to CREs at key loci of both cell-intrinsic and intercellular planar polarity proteins to shape the auditory sensory epithelium.

## DISCUSSION

During development, the spatiotemporal expression pattern of a gene is determined by its associated enhancers, which are short regulatory noncoding DNA sequences (∼100–1000-bp) with various motifs for TF binding and are the DNA platform for the recruitment of TFs and transcription regulatory machineries. Genome-wide characterization of enhancers and developmental programs that drive the generation of distinct cell types in the auditory sensory epithelium is a prerequisite to finding ways to repair it when damaged. Due to technical challenges in material collection and others, genome-wide identification of inner ear-specific enhancers has not yet been performed and no ChIP-seq data for any TFs that use inner ear sensory organs are publicly available. Previous studies on brain development discovered very few enhancers, including the 1.4-kb 3′*Atoh1* enhancer (54) and *Sox2* (55), by traditional genetic approaches through screening of DNA fragments flanking the gene bodies. In this study, the systematic mapping by Six1 ChIP-seq at different stages together with H3K27ac ChIP-seq across cell types in the cochlear epithelium provides for the first time a rich resource of sites with regulatory potential and also a ‘temporal’ clue for the activities of these sites.

Enhancer function requires binding of TFs to their motifs and cooperation between the bound TFs. This cooperativity feature of TFs enables a relatively small number of TFs to determine a large diversity of cell types, through distinct combinatorial roles of each TF (56). Motif analysis of Six1 ChIP-seq data revealed the presence of motifs for many other TFs within the sequences of Six1 CREs and both the type and percentage of enriched motifs are dynamic at different stages (Supplementary Figure S4A), suggesting that Six1 cooperates with diverse TFs to drive spatiotemporal regulatory programs during cochlear development. Among them, the RFX motifs are the most significantly enriched at both stages. Recent studies found that the Rfx family members *Rfx1, 2, 3, 5* and *7* are all detected in the hair cells during development and that RFX TFs are essential for hearing in mice (42), but the underlying mechanism is unclear. The GO analysis of the subgroup of Six1 CREs carrying RFX motifs identified ‘cilium organization’ as the most significantly enriched term, which is consistent with the known function of RFX TFs as major regulators in cilia formation (57). However, the GO terms of ‘inner ear morphogenesis and otic vesicle development’ were also identified, suggesting novel functions of the RFX as cofactors of Six1 in the inner ear. Our analyses suggest that the RFX proteins cooperate with Six1 to activate lineage-specific regulatory programs in the auditory sensory epithelium.

We found RFX motifs located adjacent to the SIX motifs with short spacing, forming a SIX:RFX motif pair, which is a common feature among TFs with direct combinatorial function. Our coIP experiments confirmed protein complex formation between Six1 and Rfx1/3 both in the cochlea epithelium and 293 cells when coexpressed (Figure 4E). Thus, it is logical to speculate that Six1 and RFX proteins form a heterodimer that binds to the SIX:RFX motif pair to exert combinatorial functions, and such combinatorial function is known to greatly increase diversity and complexity of gene regulation. This direct cooperativity was further revealed by the transgenic reporter experiment where mutation of a single SIX-motif was inadequate to abolish *Pbx1* enhancer activity, but mutation of both SIX:RFX-motifs abolished enhancer activity *in vivo* (Figure 4C). Consistent with the combinatorial function between Six1 and RFX mediated by the SIX:RFX-motifs, deletion of Six1 alone only weakened the expression of *Pbx1* in the cochlea (Figure 4D).

Besides the RFX motifs, motif analysis of Six1 CREs also revealed co-motifs for hair cell differentiation factors Gata3, Atoh1, Pou4f3 and Gfi1, all of which physically interact with Six1. Importantly, the genome-wide characterization of Six1 binding enabled us to identify previously unknown distal enhancers at the loci of *Atoh1*, Atoh1’s downstream factor *Pou4f3* and Pou4f3′s downstream factor *Gfi1*, all of which drive hair-cell-restricted expression. Based on these data, we propose that Six1 is an inner-ear sensory selector gene that sits atop the hierarchy of sequential events and engages protein complexes with downstream TFs to not only trigger cell fate induction but also regulate progressive differentiation to establish cell identity.

Selector genes are TFs that instruct the development of organs. In more recent years, the terminal selector concept has been developed to identify genes that determine specific neuron types in differentiated cells (58). The *Drosophila* Six family Sine-oculis and its partner Eya are downstream TFs of the Pax6 eye selector genes Eyeless and Twin of eyeless of the retinal determination gene network (59). These genes cross-regulate and engage protein complexes (60). We previously demonstrated that Six1–Eya1–Sox2 synergistically activate *Atoh1* to trigger the ultimate step of hair cell fate induction based on the evidence that temporal deletion of *Six1* leads to loss of hair cells (16) and that combination of Six1-Eya1 convert nonsensory GER cells into hair cells, which requires Sox2 activity (14). Six1-binding site is ∼500-bp downstream of Sox2-binding site within the 1.4-kb 3′ *Atoh1* autoregulatory enhancer (14,61). Here we have identified three additional Six1-bound CREs of *Atoh1* and that *Atoh1*+70 000 is active in hair cells (Figure 2). However, our data also point to a role for Six1 in regulating gene repression in the progenitors, as a portion of E13.5 precursor-transient peaks completely lacked H3K27ac-deposition. For example, the precursor-transient *Atoh1*+53 500 is lost by E16.5 and is inactive in differentiating hair or supporting cells, while the Six1-bound proximal-promoter CRE was previously shown to interact with the Notch mediator Hey/Hes repressor to select supporting cell fate (36). Thus, the distinct Six1-bound CREs likely mediate ‘passive’ and ‘active’ roles of Six1 in enhancing the temporal and cell-type-specific activation of *Atoh1*.

While it is currently unclear whether Six1 also requires Eya1 activity to coregulate distinct lineage-specific programs during terminal differentiation, the identification of Six1-bound sites at loci of hair-cell- or supporting-cell-specific TFs, signaling pathways, and effector genes indicates that Six1 induces sequential activation of a subset of genes that allow the formation of specialized protein complexes, which in turn activate progressively refined gene expression programs to mediate sensory epithelium patterning and growth. For instance, Six1-induced expression of Atoh1 is required for expression of many later genes, including Pou4f3 or Gfi1, that form protein complexes to jointly mediate progressive hair cell differentiation. Similarly, Six1-RFX-Pbx1 also act in a positive feedforward loop to regulate gene expression at different time points during development. Our data of a range of hair-bundle defects in *Six1^Cko/Cko^* and Six1-occupancy at loci of a wide range of regulators, PCP-signaling, and cell-type-specific effectors such as *Fgf8*/*Calb2*, *Slc26a5* and *Dusp6/S100a/Slc1a3* suggest that Six1 may function with a variety of signaling inputs to coregulate stage- and cell-type-specific gene expression. Thus, it is plausible to speculate that Six1 is also a terminal selector that determines distinct cell-types and maintains the stable identity of nondividing differentiated cells throughout life. Six1 in cooperation with different signaling molecules may be used to reiteratively cross-regulate and maintain its own expression to act as terminal selector. Consistent with this view, we have identified multiple Six1-bound sites within its own locus (Supplementary Figure S2) and observed persistent Six1 expression in the organ of Corti in adult cochlea. These Six1-binding sites may directly respond to both Six1 and signaling inputs to ensure that Six1 expression is maintained in specific cell-types.

How does Six1 act to achieve the purpose of selector genes? We find that Six1 occupies enhancers before transcription of target genes. This early action of Six1 raises the question of whether Six1 functions as a pioneer factor for hair cell differentiation. Pioneer factors, considered as a special class of embryonic master regulators with the unique ability to occupy their target sites in chromatin in a cell-specific matter, can play both ‘passive’ and ‘active’ roles in enhancing transcription (62). Passively, like other pioneer factors, Six1 may simply engage target sites in chromatin to limit their binding to other TFs later to ‘prime’ the enhancer for rapid and synchronous activation in response to developmental cues. Pioneer factors can also function actively by helping to open or organize the local chromatin, which allows it to bind to other TFs, chromatin modifiers, and coregulators (63). While future experiments are necessary to determine whether Six1 has intrinsic activity to open and organize chromatin, as discussed earlier, our data suggest that Six1 may actively help open chromatin via interaction with Brg1–BAF chromatin-remodeling complexes. The discovery of motifs in Six1 CREs for Forkhead, GATA, SOX and other factors that are known ‘active’ pioneer factors with intrinsic chromatin opening activity (63) suggests that Six1 may use the pioneering activity of these cofactors to promote local chromatin decondensation. Six1 may also have a role in mediating chromatin looping and nuclear organization through interaction with CTCF, which is one of the most enriched motifs in Six1 peaks (Figure 3).

Finally, our finding that persistent peaks are highly correlated with H3k27ac-enrichment support the notion that these are active CREs from E13.5. As Six1-bound CREs are present at many loci that encode proteins responsible for different forms of deafness syndromes, including Connexin 26 (*Gjb2*) and Pendrin (*Slc26a4*) (Supplementary Figure S7D), whose mutations are linked to ∼50% of congenital hearing loss (64,65), our study could shed new light on pathological mechanisms initiated by misregulation of these critical CREs.

## DATA AVAILABILITY

The ChIP-seq data reported in this paper were deposited to the Gene Expression Omnibus (GEO) (GSE108130 and GSE119545).

We have two GEO associated with this project. GSE108130, token is ehmxuaqqrbwphuv: https://www.ncbi.nlm.nih.gov/geo/query/acc.cgi?acc=GSE108130.

GSE119545, token is ytmxskuwrbwzncb: https://www.ncbi.nlm.nih.gov/geo/query/acc.cgi?acc=GSE119545.

## Supplementary Material

gkaa012_Supplemental_Files
