# Supplementary material for: Dynamic changes in cis-regulatory occupancy by Six1 and its cooperative interactions with distinct cofactors drive lineage-specific gene expression programs during progressive differentiation of the auditory sensory epithelium
Source: Nucleic Acids Res. 2020 Jan 20;48(6):2880–96. doi: 10.1093/nar/gkaa012 (PMC7102962; doi:10.1093/nar/gkaa012)
Supplement: gkaa012_Supplemental_Files [file gkaa012_supplemental_files.zip › Supplementary Figures-binder.pdf]

Supplementary Figure legends

Supplementary Figure S1. Genomic Six1 binding shifts between E13.5-16.5. (A) Heatmap of Six1 peaks showing fold-change of Six1-enrichment. **(B)** Selected GO terms. *P*-value of each term is shown. **(C)** Great analysis showing distance of Six1 peaks to TSS (kb). **(D,E)** Distribution (**D**) and distance to TSSs (**E**) of overlapping peaks between Six1 and H3K27ac. Note that H3k27ac was only performed in E13.5 cochleae.

Supplementary Figure S2. Genomic browser visualization of Six1 peaks illustrate the dynamic stage-related changes of Six1 binding at several key loci that encode transcription factors and Fgf signaling molecules that are essential for sensory epithelium development and cell fate selection. Four persistent peaks (purple boxes), a differentiation (green box) and a precursor-transient peak (red box) at *Hes1*; multiple persistent/differentiation peaks (stronger at E16.5) at *Fgfr1/3/Fgf9*; two persistent peak (purple boxes) and a differentiation peak (green box) at *Fgf8*; Multiple persistent (purple boxes) and differentiation peaks (green boxes) at *Six1* locus; a precursor-transient peak at 3' *Sox4*; and both persistent (purple boxes) and precursor-transient (red boxes) peaks at conserved regions (cons.) of *Pax2*. Six1-occupancy to the transient peaks at these gene loci suggests that Six1 regulates these factors in the progenitors. The multiple persistent and differentiation peaks at its own locus suggests that they may mediate autoregulation to maintain its own expression throughout sensory epithelium development. Asterisk (*) indicates a nonspecific peak at the *Fgf8* locus as it is also in IgG and input. y-Axis numerical values in each track indicate track height scaling in read depth. The direction of transcription is shown by the arrow beginning at the TSS.

Supplementary Figure S3. Six1 occupies distal enhancers of Atoh1, Pou4f3 and Gfi1 to mediate progressive hair cell differentiation. **(A-C)** G0 transgenic analysis of Six1-bound distal enhancers of *Atoh1* (**A**), *Pou4f3* (**B**) and *Gfi1* (**C**) showing HC-restricted activity in all inner ear sensory organs. **(D)** Genomic browser visualization of Six1 peaks at *Gata3*, and ChIP-qPCR analysis of 3/4 boxed peaks showing stage-related changes in Six1-binding. *P<0.05, **P<0.01. **(E)** Co-immunoprecipitation analysis of 293 cells cotransfected with *Flag-Gata3/His-Six1* expression plasmids. **(F)** Genomic browser visualization of Six1 peaks at the IHC-specific *Calb2* (Calretin), OHC-specific *Slc26a5* (Prestin), *S100a* (IHC, inner-phalangeal/Dieters' SCs) and *Slc1a3* (GLAST, inner-phalangeal/inner-border SCs) from the precursor stage. y-Axis numerical values in each track indicate track height scaling in read depth. The direction of transcription is shown by the arrow beginning at the TSS or at the TTS (transcription termination site) for *Slc1a3*.

Supplementary Figure S4. Significant motifs in Six1 peaks and Six1 interacts with RFX proteins to cooperatively regulate *Pbx1* activity in hair cells. **(A)** List of additional motifs enriched in Six1 peaks identified by motif analysis from Homer Known motifs. **(B)** Six1 occupies proximal-promoter of *Rfx1* at both stages and of *Rfx3* at E16.5. **(C)** In situ hybridization showing *Pbx1* mRNA in E10.5 otocyst (ov). Arrow points to signal in the dorsal region. **(D)** G0 transgenic analysis of Six1-bound *Pbx1*+490000 showing activity in the otocyst on sections of two independent transgenic embryos (*n*=2/2 transgenic embryos). **(E-G)** Images of whole-embryos at E10.5 showing enhancer

activity in the ventral region of the otocyst (**E**) and mutation of the Six1/2-binding motifs (SIXmt) alone did not disrupt enhancer activity in the otocyst (**F**), but mutation of both SIX:RFX motifs in combination completely disrupted enhancer activity (**G**). (H,I) Images of saccule sections showing *Pbx1*+49000 activity in both HCs and SCs (**H**) and the SIXmt only weakened the enhancer activity, particularly in HCs (**I**). **(J)** Mutation of both SIX:RFX motifs disrupted enhancer activity in the epithelium on the floor of the cochlea duct, but some $LacZ^{+}$ cells were only observed in a region above the GER (arrow).

Supplementary Figure S5. Six1-bound intronic *Dusp6*+2200 enhancer activity is restricted to *Dusp6*-expressing cells. (A) In situ hybridization showing *Dusp6* expression in otocyst (ov) at E10.5. **(B)** X-gal staining of G0 transgenic embryo at E10.5 showing *Dusp+2200-LacZ* transgene expression in the otocyst (ov) either by whole-mount view (left panel) or section (right panel) (*n*=6/6 transgenic E10.5 embryos). Note that this enhancer is not active in forelimb (fl) or hindlimb (hl) where *Dusp6* is expression (**A**). **(C)** X-gal staining of whole inner-ear showing *Dusp6+2200-LacZ* expression in all sensory epithelia at E18.5. **(D)** In situ showing *Dusp6* expression in the pillar-cells (PCs) of E17.5 $Eya1^{CreER}$ littermate cochlea, but significantly reduced expression in $Six1^{Cko/Cko}$ cochlea (tamoxifen given at E12.5). Arrows point to residual expression in the apical end of the cochlea duct in the mutant.

Supplementary Figure S6. Six1 occupies a wide range of loci that are necessary for hair-bundle morphogenesis and orientation. (A) 80.85% of over 2101 SNPs that overlapped with the 183 Six1 peaks mapped to 83 known deafness-causing genes in

humans collected in the Deafness Variant Database[1] belong to the group of unknown significance. **(B)** Genomic browser visualization of Six1 peaks at hair-bundle-related genes *Myh9*, *Clic5* and *Espn* and PCP-related genes *Celsr1, Vangl1* and *Ptk7*.

Supplementary Figure S7. Six1 regulates primary hair-bundle- and PCP-related genes and occupies key loci that are responsible for the widely distributed congenital hearing loss. (A) Immunostaining for Clic5 showing localization in hair-bundle in wild-type control and reduced expression in *Six1* CKO cochlea at P0. (**B**) qRT-PCR of E18.5 cochlea. qPCR was performed in triplicate and repeated three times. (**C**) G0 transgenic analysis of *Vangl2*+102000 enhancer showing activity in the medial region of the cochlear epithelium and all vestibular sensory epithelium. **(D)** Genomic browser visualization of Six1 peaks at the loci of *Gjb2* (Connexin26) and *Slc26a4* (Pendrin), mutations of which are linked to ~50% of congenital hearing loss[2,3]. y-Axis numerical values in each track indicate track height scaling in read depth. The direction of transcription is shown by the arrow beginning at the TSS.

1 Azaiez, H. *et al.* Genomic Landscape and Mutational Signatures of Deafness-Associated Genes. *Am J Hum Genet* **103**, 484-497, doi:10.1016/j.ajhg.2018.08.006 (2018).

2 Sheffield, A. M. & Smith, R. J. H. The Epidemiology of Deafness. *Cold Spring Harb Perspect Med*, doi:10.1101/cshperspect.a033258 (2018).

3 Yu, H., Liu, D., Yang, J. & Wu, Z. Prevalence of mutations in the GJB2, SLC26A4, GJB3, and MT-RNR1 genes in 103 children with sensorineural hearing loss in Shaoxing, China. *Ear Nose Throat J* **97**, E33-E38, doi:10.1177/014556131809700603 (2018).

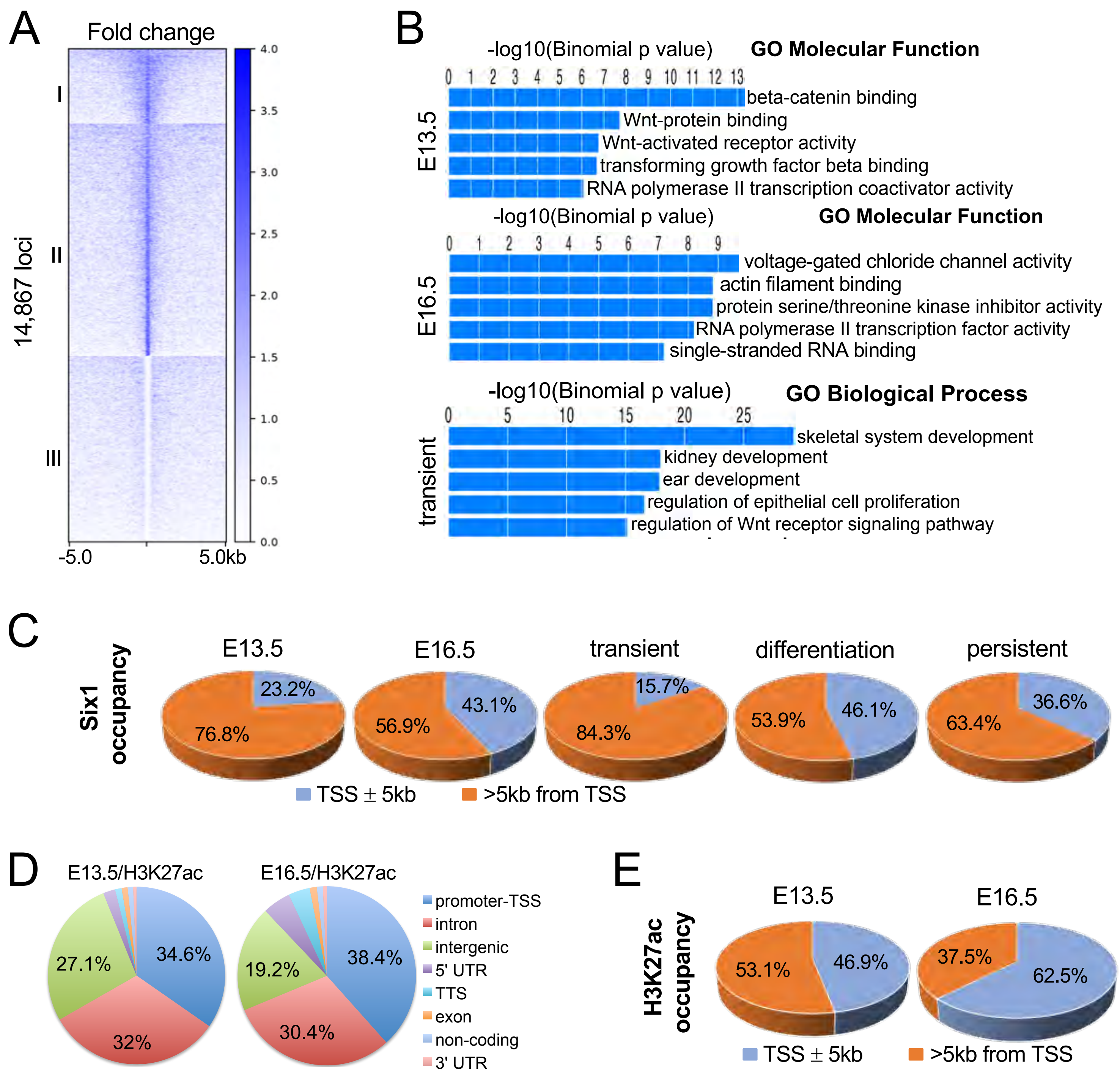
A
Fold change
I
II
III
14,867 loci
-5.0
5.0kb
4.0
3.5
3.0
2.5
2.0
1.5
1.0
0.5
0.0
B
-log10(Binomial p value)
GO Molecular Function
E13.5
beta-catenin binding
Wnt-protein binding
Wnt-activated receptor activity
transforming growth factor beta binding
RNA polymerase II transcription coactivator activity
-log10(Binomial p value)
GO Molecular Function
E16.5
voltage-gated chloride channel activity
actin filament binding
protein serine/threonine kinase inhibitor activity
RNA polymerase II transcription factor activity
single-stranded RNA binding
-log10(Binomial p value)
GO Biological Process
transient
skeletal system development
kidney development
ear development
regulation of epithelial cell proliferation
regulation of Wnt receptor signaling pathway
C
Six1 occupancy
E13.5
23.2%
76.8%
E16.5
43.1%
56.9%
transient
15.7%
84.3%
differentiation
53.9%
46.1%
persistent
36.6%
63.4%
TSS ± 5kb
>5kb from TSS
D
E13.5/H3K27ac
34.6%
27.1%
32%
E16.5/H3K27ac
38.4%
19.2%
30.4%
promoter-TSS
intron
intergenic
5' UTR
TTS
exon
non-coding
3' UTR
E
H3K27ac occupancy
E13.5
53.1%
46.9%
E16.5
37.5%
62.5%
TSS ± 5kb
>5kb from TSS

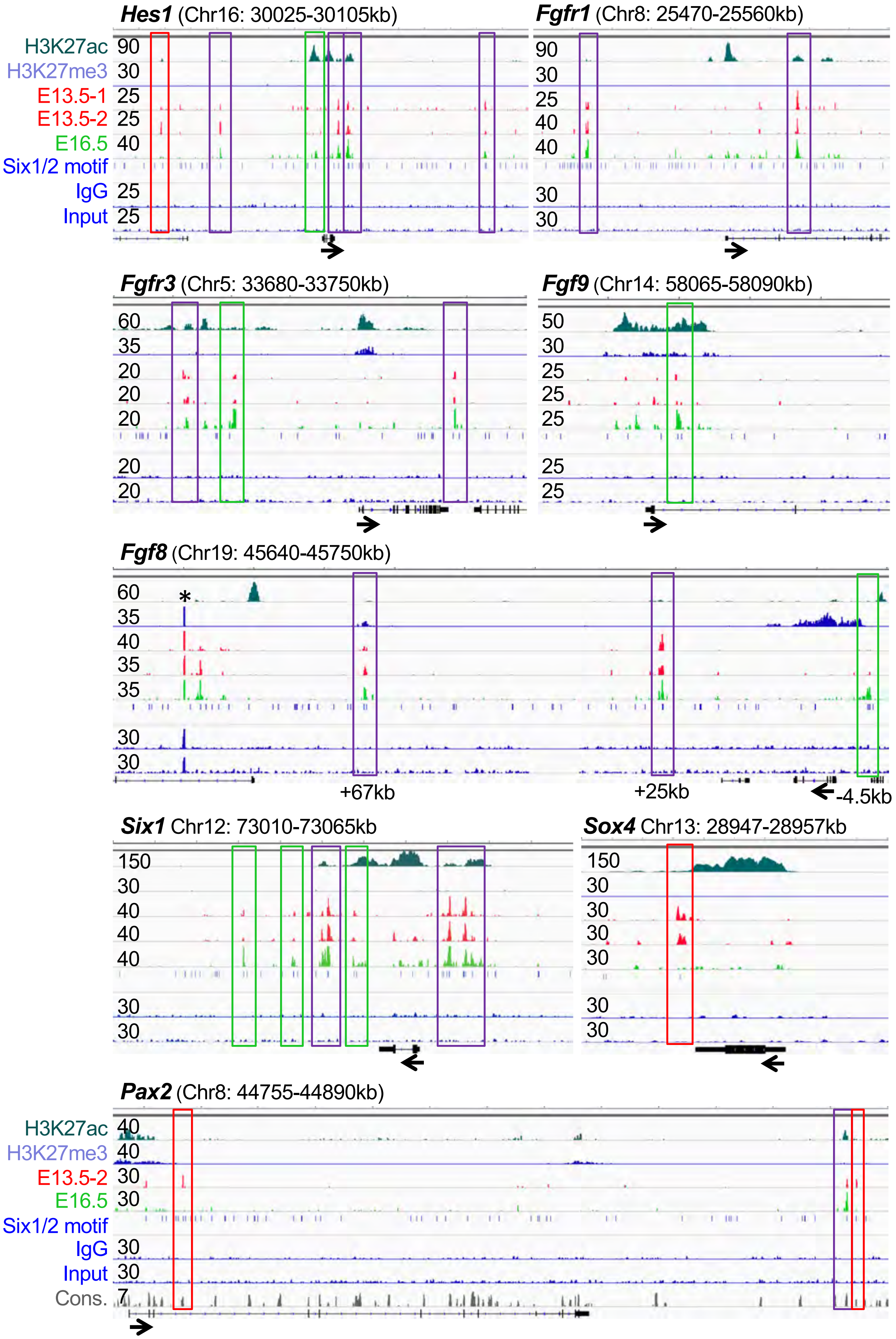
Hes1 (Chr16: 30025-30105kb)
Fgfr1 (Chr8: 25470-25560kb)
H3K27ac
H3K27me3
E13.5-1
E13.5-2
E16.5
Six1/2 motif
IgG
Input
Fgfr3 (Chr5: 33680-33750kb)
Fgf9 (Chr14: 58065-58090kb)
Fgf8 (Chr19: 45640-45750kb)
+67kb
+25kb
-4.5kb
Six1 Chr12: 73010-73065kb
Sox4 Chr13: 28947-28957kb
Pax2 (Chr8: 44755-44890kb)
Cons.

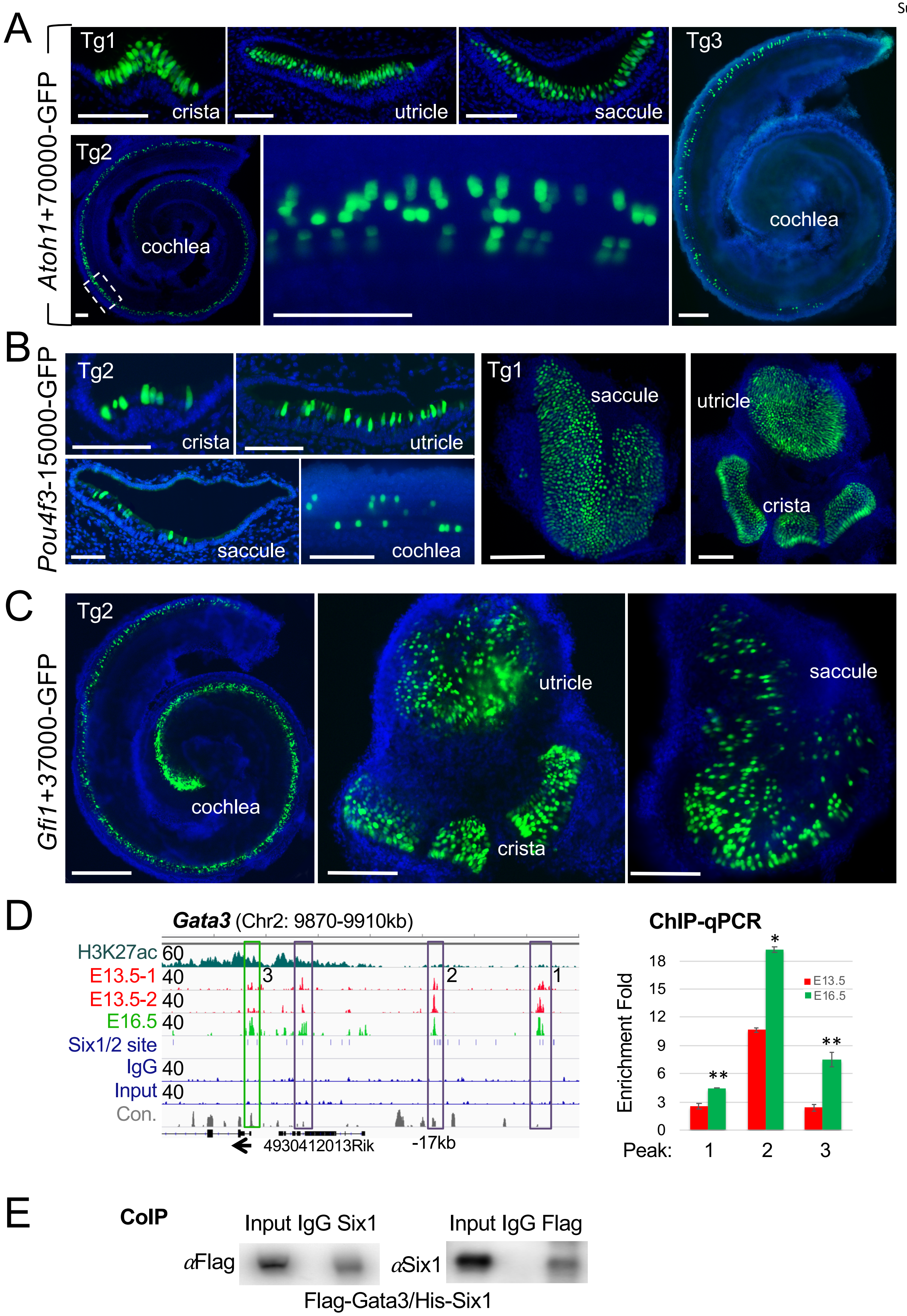
A
Atoh1+70000-GFP
Tg1
crista
utricle
saccule
Tg3
cochlea
Tg2
cochlea
B
Pou4f3-15000-GFP
Tg2
crista
utricle
saccule
cochlea
Tg1
saccule
utricle
crista
C
Gfi1+37000-GFP
Tg2
cochlea
utricle
crista
saccule
D
Gata3 (Chr2: 9870-9910kb)
H3K27ac 60
E13.5-1 40
E13.5-2 40
E16.5 40
Six1/2 site
IgG 40
Input 40
Con.
3
2
1
4930412013Rik
-17kb
ChIP-qPCR
Enrichment Fold
E13.5
E16.5
Peak: 1 2 3
E
CoIP
Input IgG Six1
Input IgG Flag
αFlag
αSix1
Flag-Gata3/His-Six1

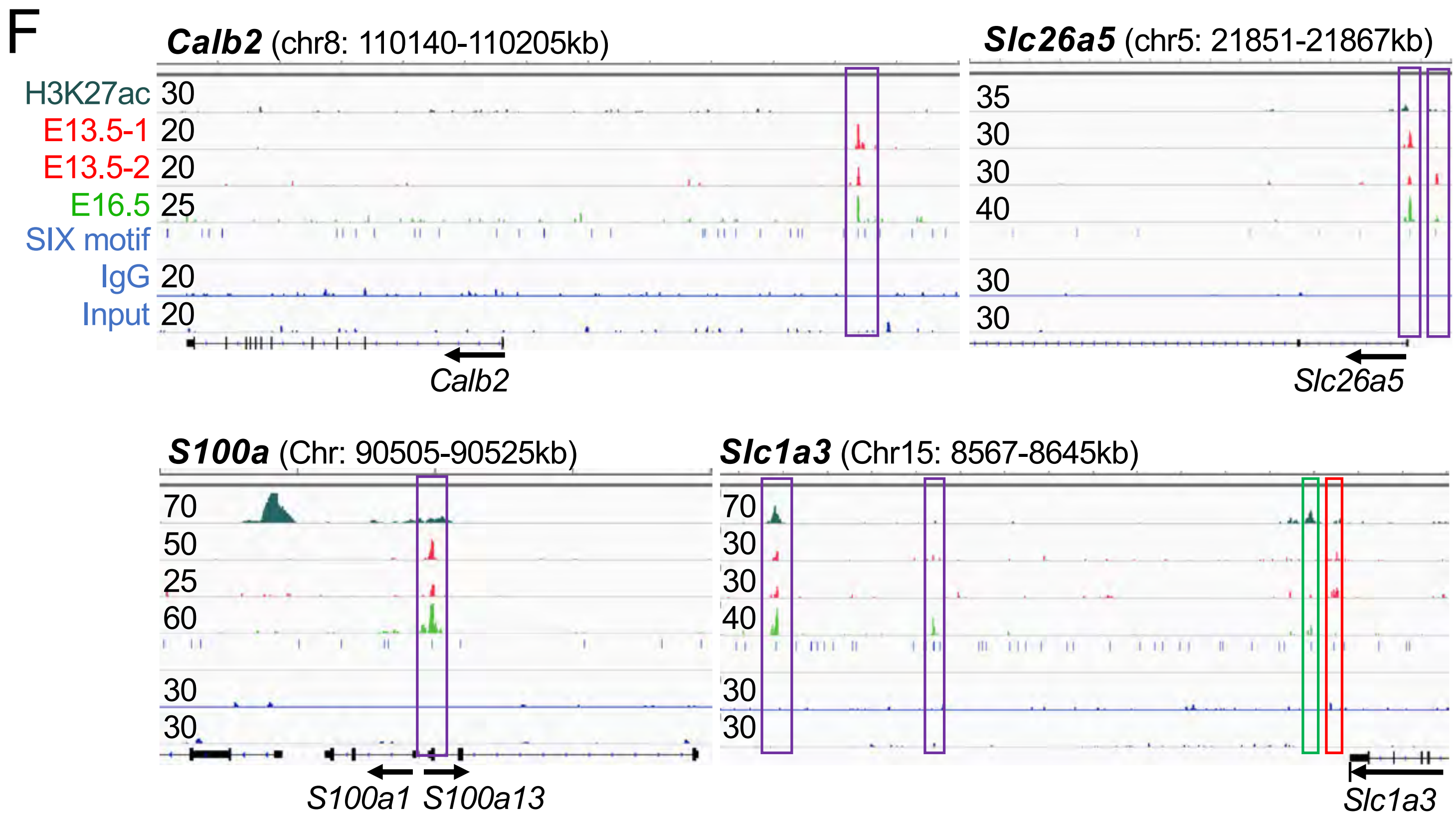
F
Calb2 (chr8: 110140-110205kb)
Slc26a5 (chr5: 21851-21867kb)
H3K27ac 30
E13.5-1 20
E13.5-2 20
E16.5 25
SIX motif
IgG 20
Input 20
35
30
30
40
30
30
Calb2
Slc26a5
S100a (Chr: 90505-90525kb)
Slc1a3 (Chr15: 8567-8645kb)
70
50
25
60
30
30
70
30
30
40
30
30
S100a1
S100a13
Slc1a3

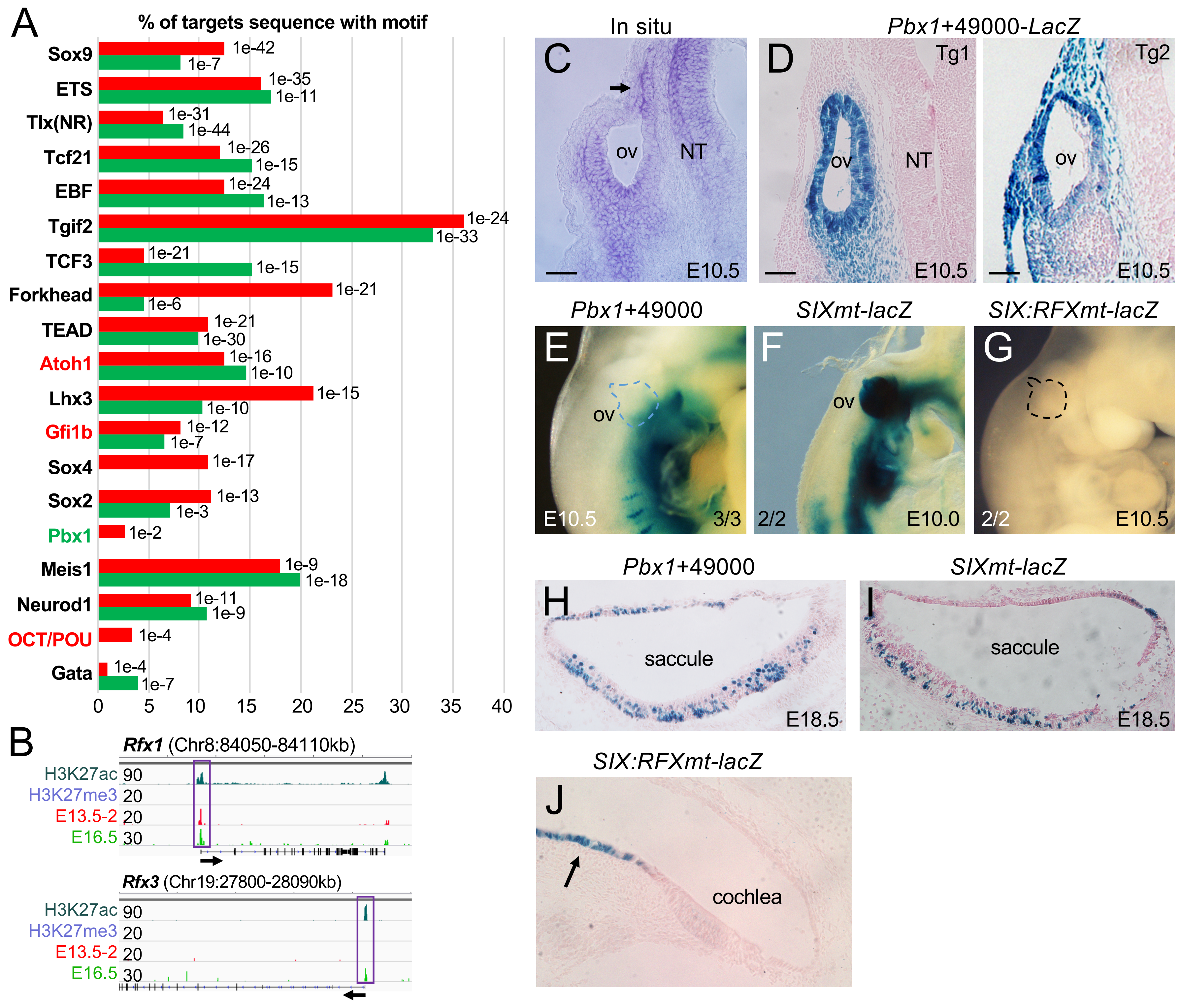
A
% of targets sequence with motif
Sox9
1e-42
1e-7
ETS
1e-35
1e-11
Tlx(NR)
1e-31
1e-44
Tcf21
1e-26
1e-15
EBF
1e-24
1e-13
Tgif2
1e-24
1e-33
TCF3
1e-21
1e-15
Forkhead
1e-21
1e-6
TEAD
1e-21
1e-30
Atoh1
1e-16
1e-10
Lhx3
1e-15
1e-10
Gfi1b
1e-12
1e-7
Sox4
1e-17
Sox2
1e-13
1e-3
Pbx1
1e-2
Meis1
1e-9
1e-18
Neurod1
1e-11
1e-9
OCT/POU
1e-4
Gata
1e-4
1e-7
0 5 10 15 20 25 30 35 40
B
Rfx1 (Chr8:84050-84110kb)
H3K27ac 90
H3K27me3 20
E13.5-2 20
E16.5 30
Rfx3 (Chr19:27800-28090kb)
H3K27ac 90
H3K27me3 20
E13.5-2 20
E16.5 30
In situ
C
ov
NT
E10.5
Pbx1+49000-LacZ
D
Tg1
ov
NT
E10.5
Tg2
ov
E10.5
Pbx1+49000
E
ov
E10.5
3/3
SIXmt-lacZ
F
ov
2/2
E10.0
SIX:RFXmt-lacZ
G
2/2
E10.5
Pbx1+49000
H
saccule
E18.5
SIXmt-lacZ
I
saccule
E18.5
SIX:RFXmt-lacZ
J
cochlea

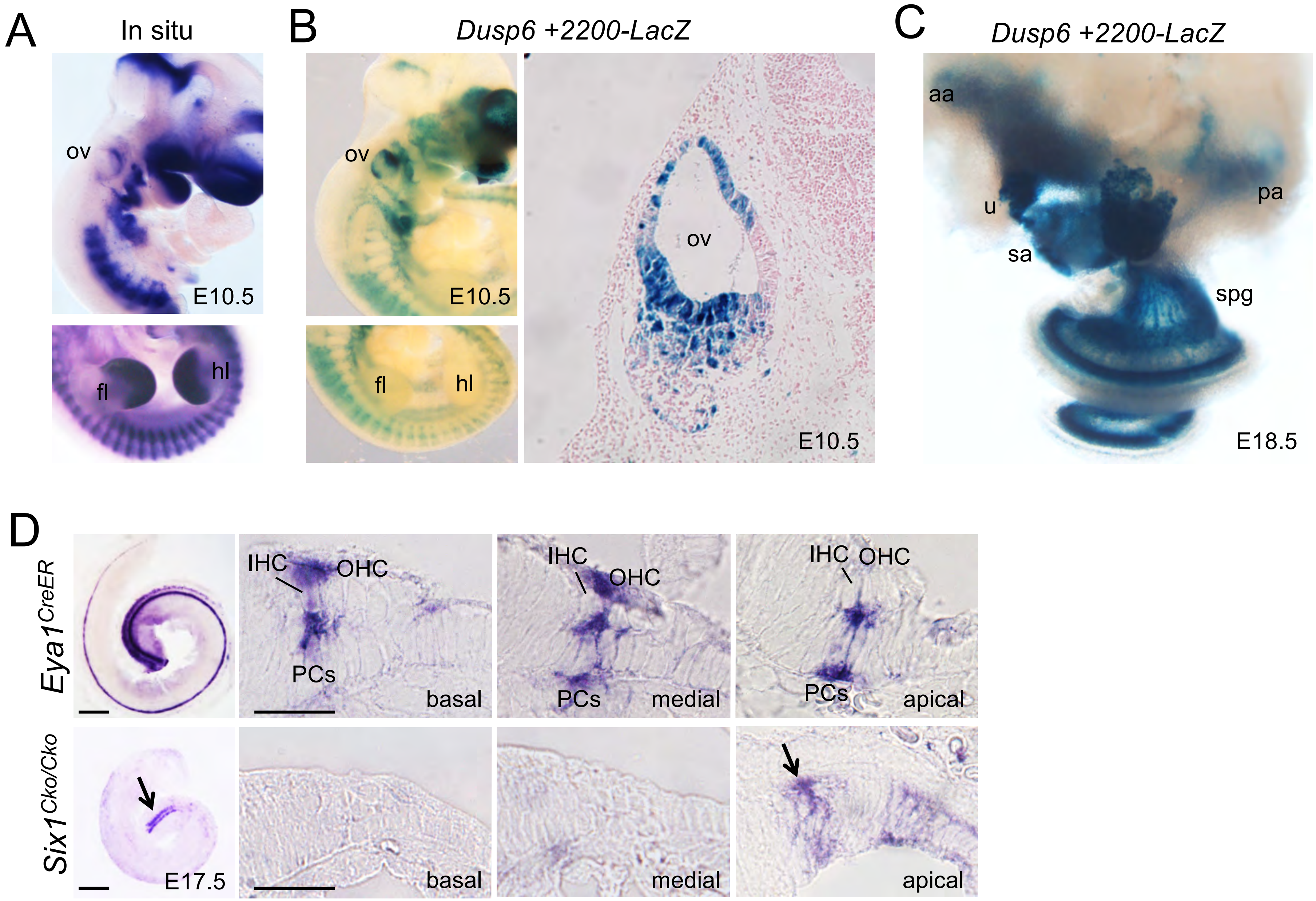
A
In situ
ov
E10.5
fl
hl
B
Dusp6 +2200-LacZ
ov
E10.5
fl
hl
ov
E10.5
C
Dusp6 +2200-LacZ
aa
u
pa
sa
spg
E18.5
D
*Eya1*CreER
IHC
OHC
PCs
basal
IHC
OHC
PCs
medial
IHC
OHC
PCs
apical
*Six1*Cko/Cko
E17.5
basal
medial
apical

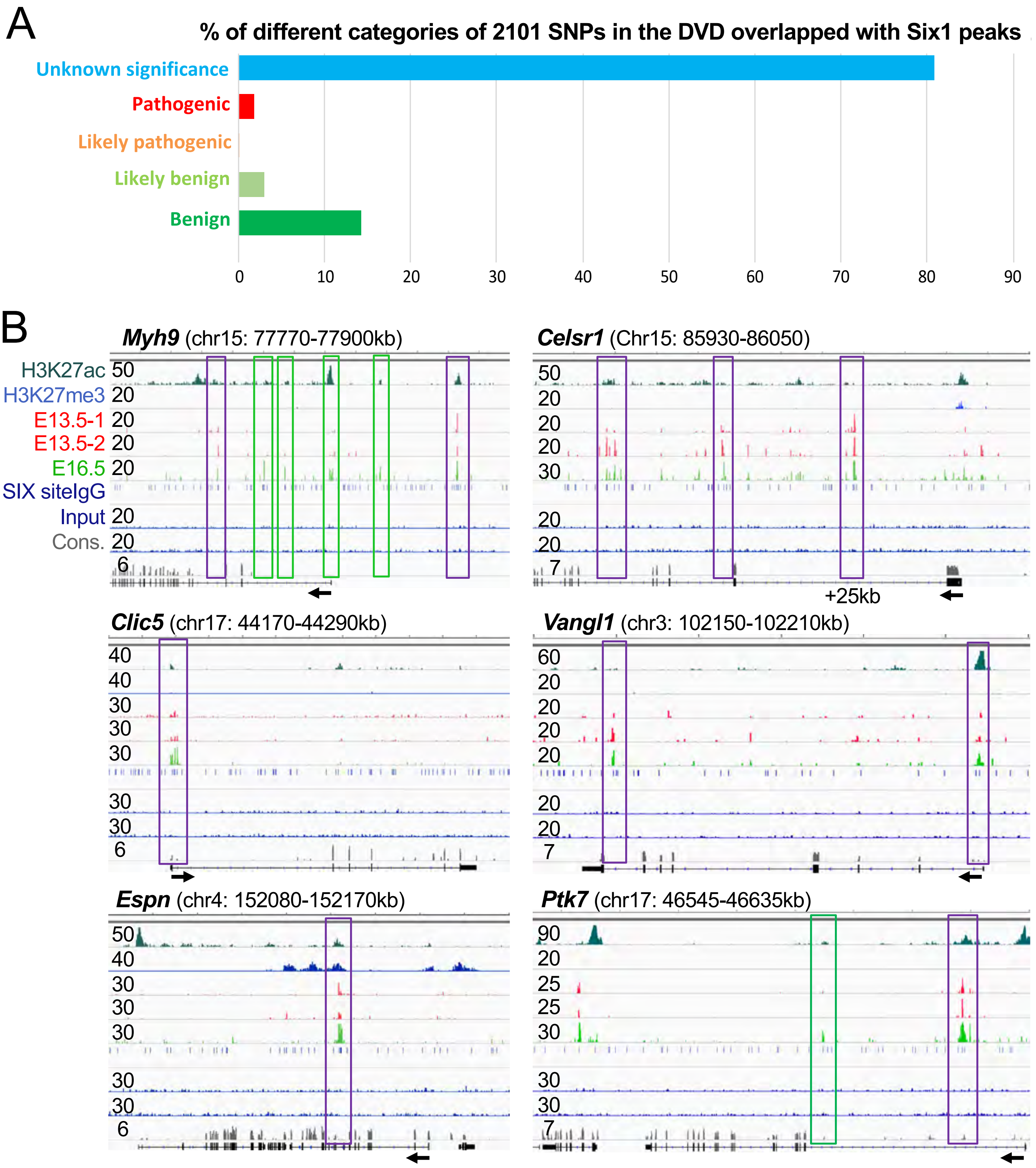
A
% of different categories of 2101 SNPs in the DVD overlapped with Six1 peaks
Unknown significance
Pathogenic
Likely pathogenic
Likely benign
Benign
0 10 20 30 40 50 60 70 80 90
B
Myh9 (chr15: 77770-77900kb)
Celsr1 (Chr15: 85930-86050)
H3K27ac
H3K27me3
E13.5-1
E13.5-2
E16.5
SIX siteIgG
Input
Cons.
+25kb
Clic5 (chr17: 44170-44290kb)
Vangl1 (chr3: 102150-102210kb)
Espn (chr4: 152080-152170kb)
Ptk7 (chr17: 46545-46635kb)

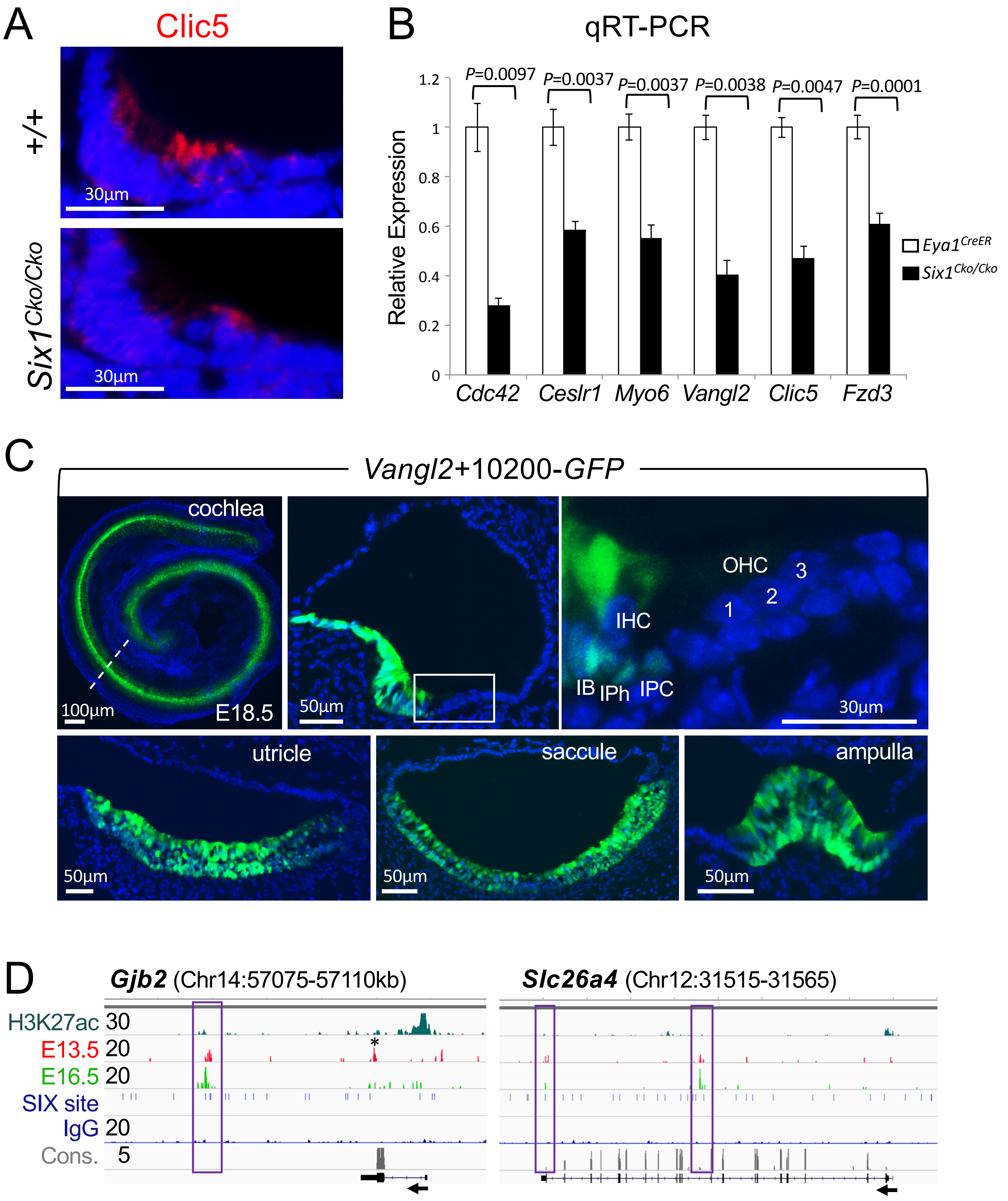
A
Clic5
+/+
30µm
Six1Cko/Cko
30µm
B
qRT-PCR
Relative Expression
1.2
1
0.8
0.6
0.4
0.2
0
P=0.0097
P=0.0037
P=0.0037
P=0.0038
P=0.0047
P=0.0001
Cdc42
Ceslr1
Myo6
Vangl2
Clic5
Fzd3
Eya1CreER
Six1Cko/Cko
C
Vangl2+10200-GFP
cochlea
100µm
E18.5
50µm
OHC
3
2
1
IHC
IB
IPh
IPC
30µm
utricle
50µm
saccule
50µm
ampulla
50µm
D
Gjb2 (Chr14:57075-57110kb)
Slc26a4 (Chr12:31515-31565)
H3K27ac 30
E13.5 20
E16.5 20
SIX site
IgG 20
Cons. 5
*
